# Biofilm Bacteria Use Stress Responses to Detect and Respond to Competitors

**DOI:** 10.1016/j.cub.2020.01.065

**Published:** 2020-04-06

**Authors:** Bram Lories, Stefanie Roberfroid, Lise Dieltjens, David De Coster, Kevin R. Foster, Hans P. Steenackers

**Affiliations:** 1 Centre of Microbial and Plant Genetics (CMPG), Department of Microbial and Molecular Systems, KU Leuven, Kasteelpark Arenberg 20, 3001 Leuven, Belgium; 2 Department of Zoology, University of Oxford, Oxford OX1 3PS, UK

**Keywords:** competition, competition sensing, microbial ecology, stress response systems, *Salmonella*, biofilm, epithelial invasion, antibiotic tolerance

## Abstract

Bacteria use complex regulatory networks to cope with stress, but the function of these networks in natural habitats is poorly understood. The competition sensing hypothesis states that bacterial stress response systems can serve to detect ecological competition, but studying regulatory responses in diverse communities is challenging. Here, we solve this problem by using differential fluorescence induction to screen the *Salmonella* Typhimurium genome for loci that respond, at the single-cell level, to life in biofilms with competing strains of *S.* Typhimurium and *Escherichia coli*. This screening reveals the presence of competing strains drives up the expression of genes associated with biofilm matrix production (CsgD pathway), epithelial invasion (SPI1 invasion system), and, finally, chemical efflux and antibiotic tolerance (TolC efflux pump and AadA aminoglycoside 3-adenyltransferase). We validate that these regulatory changes result in the predicted phenotypic changes in biofilm, mammalian cell invasion, and antibiotic tolerance. We further show that these responses arise via activation of major stress responses, providing direct support for the competition sensing hypothesis. Moreover, inactivation of the type VI secretion system (T6SS) of a competitor annuls the responses to competition, indicating that T6SS-derived cell damage activates these stress response systems. Our work shows that bacteria use stress responses to detect and respond to competition in a manner important for major phenotypes, including biofilm formation, virulence, and antibiotic tolerance.

## Highlights

• *Salmonella* induces biofilm formation, invasion, and efflux in response to competition
• Traditional stress response systems regulate this competitive response
• These stress responses are activated in response to a T6SS-mediated attack
• Together, these data provide direct support for the competition sensing hypothesis

## Teaser

Bacteria typically live in dense and diverse communities; however, little is known about how bacteria respond to competing strains on a molecular level. Lories et al. report that *Salmonella* detects competitors via stress response systems and induces pathways important for biofilm formation, epithelial invasion, and antibiotic tolerance.

## Introduction

Bacteria form dense surface-associated communities, known as biofilms [1], which cause major problems and economic losses within industrial and medical sectors [2]. Within biofilms, ecological competition is often intense [3, 4], particularly among strains with overlapping nutrient requirements [5, 6, 7]. Ecological competition—hereafter “competition”—includes all negative effects of one cell on the fitness of other cells, which can either result from evolutionary adaptations to harm other genotypes or be accidental [8]. A key corollary of the importance of competition is that it should strongly shape bacterial regulatory networks. Natural selection should favor cells that can detect and respond appropriately to the harm caused by competing strains, as stated by the competition sensing hypothesis [9].

In line with this hypothesis, a number of studies subsequently showed that the presence of competitors can induce bacteria to produce more biofilm [4], change the amount of antimicrobials they secrete [10, 11], or become more tolerant toward antibiotics [12]. Although these studies show that bacteria indeed change their behavior in response to competitors, they focused on the phenotypic bases for these responses, not on the regulatory networks that are central to the competition sensing idea. One reason for this focus is the technical challenge associated with studying regulatory responses in mixed genotype biofilms. Competing bacteria are often phylogenetically similar, making it hard to distinguish between transcripts of different strains [8, 13]. Moreover, the complex, heterogeneous environment within structured communities, such as biofilms, drives strong cell-to-cell variation, meaning that transcriptional measures that average the population can miss strong effects at the single-cell level [14]. A range of emerging techniques offer solutions for studying single-cell gene expression, and one approach that circumvents both challenges is differential fluorescence induction. This method is an enrichment strategy developed for *Salmonella* Typhimurium, in which a promoter trap library—where each strain in the library contains a small section of the bacterial chromosome followed by a GFP reporter—is screened genome-wide at the single-cell level to identify genes upregulated under specific environmental conditions [15].

Here, we apply differential fluorescence induction to study how bacteria respond to the presence of competing strains in biofilms. We first established a new mixed-species biofilm model that is sufficiently complex to capture diverse effects of competition but sufficiently simple for advanced molecular methods: a mixed-species biofilm of two *S.* Typhimurium strains and one *Escherichia coli* strain. Specifically, we put a promoter trap library in *Salmonella* through a stringent selection regimen. This regimen employed flow cytometry to select for genes that were upregulated both in biofilms and in the presence of competing strains [16, 17]. In this way, we could identify single-cell regulatory responses of *Salmonella* Typhimurium to competing strains within a biofilm community.

Our approach reveals that *Salmonella* responds to the presence of competing genotypes in multiple ways, upregulating genes associated with biofilm formation, epithelial invasion, and antibiotic tolerance, along with their concomitant phenotypes. We further show that major stress response regulators are key to the way that *Salmonella* senses and responds to competing bacteria and that the action of the type VI secretion system—the poisoned molecular spear-gun carried by many gram-negative bacteria—is a key stimulus of the competitive responses. Our work shows how competition between strains can be central to the regulatory responses within mixed-species communities.

## Results

### A Competitive Mixed-Species Biofilm Model

**Figure 1:**
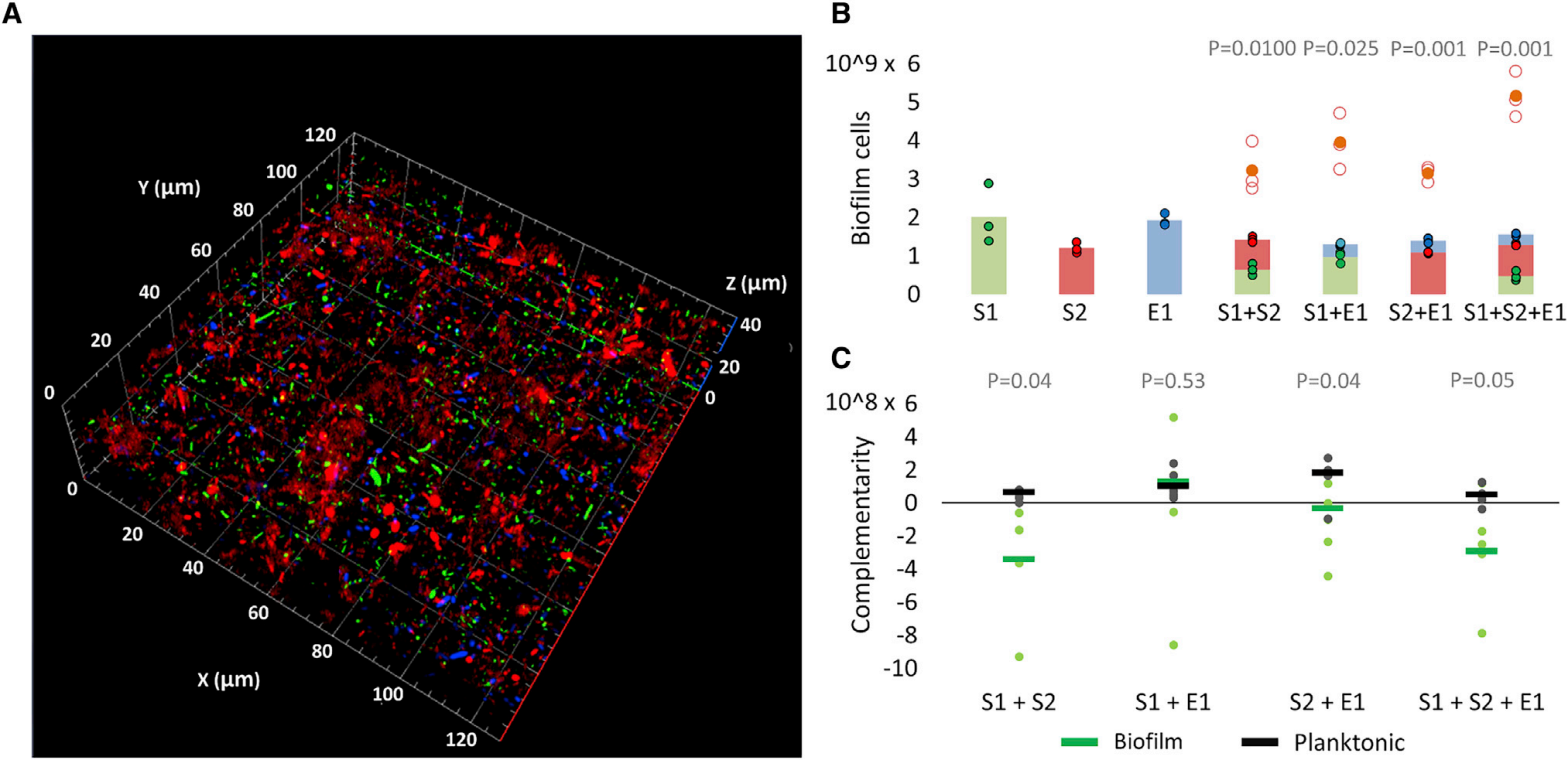
The Mixed-Species Biofilm Model Is Characterized by Competitive Interactions (A) Confocal micrograph of the mixed-species biofilm model containing wild-type *S.* Typhimurium strains S1 (green) and S2 (red) and *E. coli* strain E1 (blue). All strains are present in similar amounts, indicating the absence of competitive exclusion (Zeiss confocal laser scanning microscope [LSM 700], with digital camera [AxioCam MRm], and the associated Zen 2011 software). S1, S2, and E1 were labeled with plasmid-encoded constitutive GFPmut3, dsRed.T4, and BFP, respectively. (B) Cell number of each strain in single-strain, two-strain, and three-strain biofilms. The cell number of each strain is greatly reduced in mixed culture as compared to monoculture, indicating strong competition between the strains. The total number of cells expected for cooperation between strains is at minimum equal to the sum of the cells in monoculture and is indicated with orange circles [8]. S1 accounted for around 30% of the biofilm cells, which is sufficient for DFI analysis. Three different biological repeats and their average are shown. p values are derived from two-tailed Student’s t test using Welch’s correction if SDs are significantly (p < 0.05) different. To differentiate between the strains, S1 was labeled with constitutive GFPmut3 on a plasmid, although S2 and E1 were labeled with plasmid-encoded constitutive dsRed.T4. Differences in colony shape and size allowed differentiation between S2 and E1 during CFU counting. The fluorescent protein markers did not influence the experimental outcome (Figure S1A). (C) The complementarity effect of mixed-species cultures. The mixed-species biofilm model, as well as the pairwise combination of S1 and S2, show negative complementarity, indicating that, besides resource competition, also physical or chemical interference occurs in these communities [12, 25]. Five different biological repeats and their average are shown. p values are derived from two-tailed Student’s t test using Welch’s correction if SDs are significantly (p < 0.05) different. See also Figure S1.

We started by establishing a mixed-species biofilm model, consisting of two *S*. Typhimurium strains, SL1344 (S1) and ATCC14028 (S2), and one *E. coli* strain, MG1655 (E1). S1, a histidine auxotroph derivative of ST4/74 [17], was used because the promoter-trap library for differential fluorescence induction (DFI) screening was previously constructed with its genomic DNA [15, 16]. The two *Salmonella* strains are distinct genetically and phenotypically. For example, the S1 strain has a reduced matrix production compared to the S2 strain, due to a defective MlrA regulator and lower *csgD* levels [14, 18], whereas S2 has reduced invasion in epithelial cells due to the lack of the SPI-1 effector protein SopE [19]. The three-strain community was chosen to be sufficiently complex to capture diverse effects of intra- and interspecific competition but sufficiently simple for detailed ecological study and advanced molecular methods. In addition to tractability, studying interactions between these two species in *ex vivo* biofilms has ecological relevance because they commonly co-occur on herbs and spices, in cattle feedlots, in food processing plants [20, 21], and in fecal contaminations [22]. They also co-occur in the mammalian microbiome [23], where they are known to compete [24]. The three strains form a stable mixed biofilm. Inoculation of a 1:1:1 ratio resulted in the formation of a reproducible 35-μm biofilm after 48 h on the bottom of the Petri dish (Figure 1A). The expectation is that the strains will be competitors, as is typical for phylogenetically similar strains that meet in nature [4]. To test for competition, cellular yield (i.e., number of colony-forming units [CFUs]) of each strain was compared between the mixed-species biofilm and monoculture biofilms (with each strain having the same inoculation size in monoculture and mixed culture) [8]. This revealed strong competition, where each strain performed worse (between 29% and 89% reduction in CFUs) in the mixed biofilm than in monoculture (Figure 1B). All pairwise combinations of strains were strongly competitive in nature as well. Also in liquid culture (hereafter referred to as planktonic condition), all interactions were competitive (Figure S1B), but the growth of S1 was inhibited to a lower extent than in the mixed-species biofilm (Figure S1C).

Loreau and Hector [25] introduced a useful logic based on “complementarity” to determine which types of competition (resource versus interference competition) occur between the strains. Resource competition includes all interactions where bacteria lower the fitness of others via the consumption of common resources, although interference competition involves more direct harm, e.g., producing antibiotics [8]. The complementarity differentiates between these types of competition by assessing whether species yields in a mixture are on average higher or lower than expected, relative to the weighted average monoculture yield of the component species [12]. Positive complementarity indicates resource partitioning or facilitation, although negative complementarity indicates physical or chemical interference. Both the mixed-species biofilm model and the pairwise combination of S1 and S2 were found to show a negative complementarity, which indicates that physical or chemical interference plays a role (Figure 1C). This negative complementarity was mitigated when the strains were grown in well-shaken liquid culture, confirming that proximity of the bacteria within the biofilm considerably enhances competition.

### DFI Screening for Genes Upregulated under Competition

**Figure 2:**
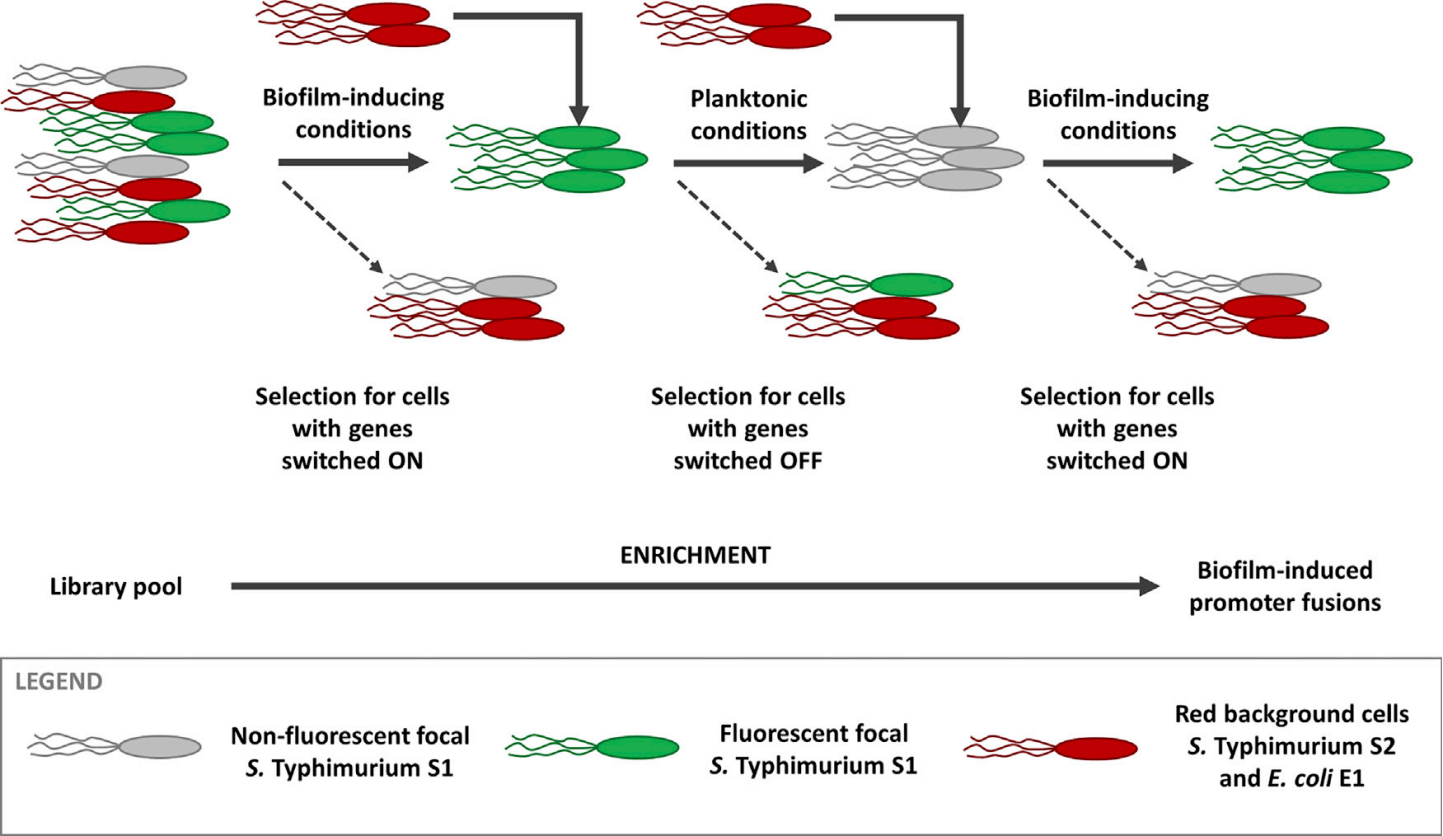
Differential Fluorescence Induction (DFI) Enriches for Promoters Specifically Expressed in the Mixed-Species Biofilm Model The DFI protocol allows to enrich the *Salmonella* Typhimurium SL1344 (S1) promoter trap library (20,500 *gfp*mut3 fusions) for promoters specifically expressed in the mixed-species biofilm model. S1 wild-type cells were alternatively subjected to biofilm-inducing and planktonic mixed-species growth conditions. In a first positive selection step, S1 was grown in mixed-species biofilm conditions, where only S1 cells with higher green fluorescence compared to a pre-determined biologically relevant threshold value were sorted with FACS. The subpool of sorted cells was subsequently amplified by overnight growth in lysogeny broth and subjected to mixed-species planktonic conditions to exclude constitutively expressed genes from the subpool. In this negative selection round, only non-fluorescent cells were sorted and amplified. A second positive selection round in mixed-species biofilm conditions resulted in a final subpool of S1 cells with plasmids containing DNA fragments with promoters that are specifically switched on in the mixed-species biofilm compared to mixed-species planktonic conditions. See also Figure S2.

Our goal is to identify robust regulatory responses to competing strains and species. To do this, we performed a genome-wide screening by DFI for genes in our focal *S.* Typhimurium (S1) upregulated in the mixed-species biofilm model. The study of social interactions in biofilms is a novel application of DFI, as previous work has focused on virulence-related genes in non-biofilm models [26]. DFI utilizes a promoter trap library constructed by cloning random fragments of bacterial genomic DNA upstream of a promoterless *gfpmut3* in plasmid pFPV25 and transforming these plasmids to S1. The library consists of approximately 20,500 different clones and was divided in 21 pools of ∼1,000 clones each prior to DFI analysis. S2 and E1 were both labeled with plasmid-encoded constitutive *dsRed.T4*. As illustrated in Figure 2, we used the DFI protocol to enrich the pools for promoters specifically expressed in the mixed-species biofilm model by alternating biofilm and planktonic conditions that respectively select (by fluorescence-activating cell sorting [FACS]) green fluorescent and non-fluorescent cells.

Cycling between the three-strain community in a biofilm and in planktonic culture allows us to select for promoters highly expressed under strong competition (biofilm) and against promoters expressed under weak competition (planktonic). Because this analysis is done at the single-cell level, we will not lose promoters that are only highly expressed in a subpopulation of the mixed-species biofilm. A limitation of this approach is that it will also select responses to biofilm formation that are not related to the presence of competing genotypes. We solve this with a second manipulation using defined promoter fusions that isolate the responses to mixed culture (next section). An alternative approach would be to directly compare mixed biofilms with monospecies biofilms. However, here, the initial negative selection step of DFI would exclude genes that are active in monospecies biofilms and limit the ability to detect the effects of competition on biofilm formation itself [4].

The heterogeneity of cells in biofilms, combined with diverse effects of strain-strain interactions, raises the possibility of considerable variability and complexity in any responses and therefore a large number of false positives. We therefore employed a stringent selection that identifies genes showing a net differential response of a stable GFP fusion across a 48-h period in each cycle of selection and then we performed two cycles of positive and one cycle of negative selection. This stringency means one only expects to identify relatively few hits at the end of the experiment.

After plating out the DFI-enriched pools, 96 clones per pool (2,016 in total) were isolated and grown separately both in mixed-species biofilm and planktonic conditions, and the fluorescence under each condition was measured at the single-cell level by flow cytometry. For each clone, the flow cytometry profiles (population distribution of fluorescence) of biofilm and planktonic conditions were compared by probability binning to exclude false positives. This method, based on a Cox chi-square test, quantitatively compares the different expression profiles and determines significant differences based on a biological relevant threshold value (STAR Methods; Figure S2A) [27]. By analyzing single clones, we remove all genes that do not differ in expression between the different conditions but were solely selected due to noisy or bimodal expression profiles [28]. The promoters upregulated under mixed-species biofilm conditions were sequenced. The activity of the corresponding genes was confirmed using defined promoter *gfpmut3* fusions because one cannot exclude non-specific effects during library construction with random S1 genomic fragments (STAR Methods). This strategy resulted in a final list of thirteen genes that are upregulated in the mixed-species biofilm compared to mixed-species planktonic conditions (Table 1).

**Table 1:** *S.* Typhimurium SL1344 (S1) Promoter Regions Induced in Mixed-Species Biofilm Compared to Mixed-Species Planktonic State

Sequence Reading into *gfpmut3*	Gene Function^a^	Gene Identifier^b^
*sitA* promoter	iron transport protein; periplasmic-binding protein; fur-regulated	STM2861
*exbB* promoter	TonB-dependent energy transduction system; fur-regulated	STM3159
*yciU* promoter	conserved hypothetical protein	STM1740
*fhuA* promoter	ferrichrome-iron receptor; fur-regulated	STM0191
*ybdO* promoter	hypothetical LysR-family transcriptional regulator	STM0606
*nrdH* promoter	hypothetical glutaredoxin	STM2805
*yeeF* promoter	hypothetical amino acid transporter protein	STM2068
*guaC* promoter	GMP reductase stringent response	STM0141
*aadA* promoter	aminoglycoside adenyltransferase; involved in aminoglycoside resistance	STM1264
*invF* promoter	AraC-family regulatory protein	STM2899
*hilC* promoter	AraC-family transcriptional regulator	STM2867
*csgB* promoter	nucleation component of curli monomers	STM1143
*tolC* promoter	outer membrane porin; outer membrane component of several multi-drug efflux systems	STM3186

a Function of the identified upregulated genes according to GenBank [29].

b STM gene number of the identified gene in *S*. Typhimurium LT2. See also Figure S3.

### Some Genes Respond to Biofilm Formation and Not Competition

The induced expression of the identified genes in the mixed-species biofilm can be a consequence of mixed culture or the biofilm mode of life. To distinguish between these alternatives, we measured the expression of the thirteen promoter GFP fusions (Table 1) under four conditions: monospecies biofilm; monospecies planktonic; mixed-species biofilm; and mixed-species planktonic.

Eight of thirteen genes showed a similar induction upon biofilm formation in monospecies and mixed-species conditions compared to planktonic conditions and a similar expression in mono- and mixed-species biofilms (Figure S3). Among these genes are the iron transport genes *sitA*, *fhuA*, and *exbB*; the GMP reductase gene *guaC*; and a number of less well-characterized genes (*yciU*, *ybdO*, *nrdH*, and *yeeF*). Importantly, three of these genes (*sitA*, *fhuA*, and *nrdH*) were also identified in previous work that used DFI to study genes upregulated in monospecies biofilms compared to planktonic conditions [16]. To focus on mixed culture and competition, however, we excluded these eight biofilm-associated genes from further analysis.

### Competition Is Associated with Genes for Biofilm Formation, Epithelial Invasion, and Antibiotic Tolerance

**Figure 3:**
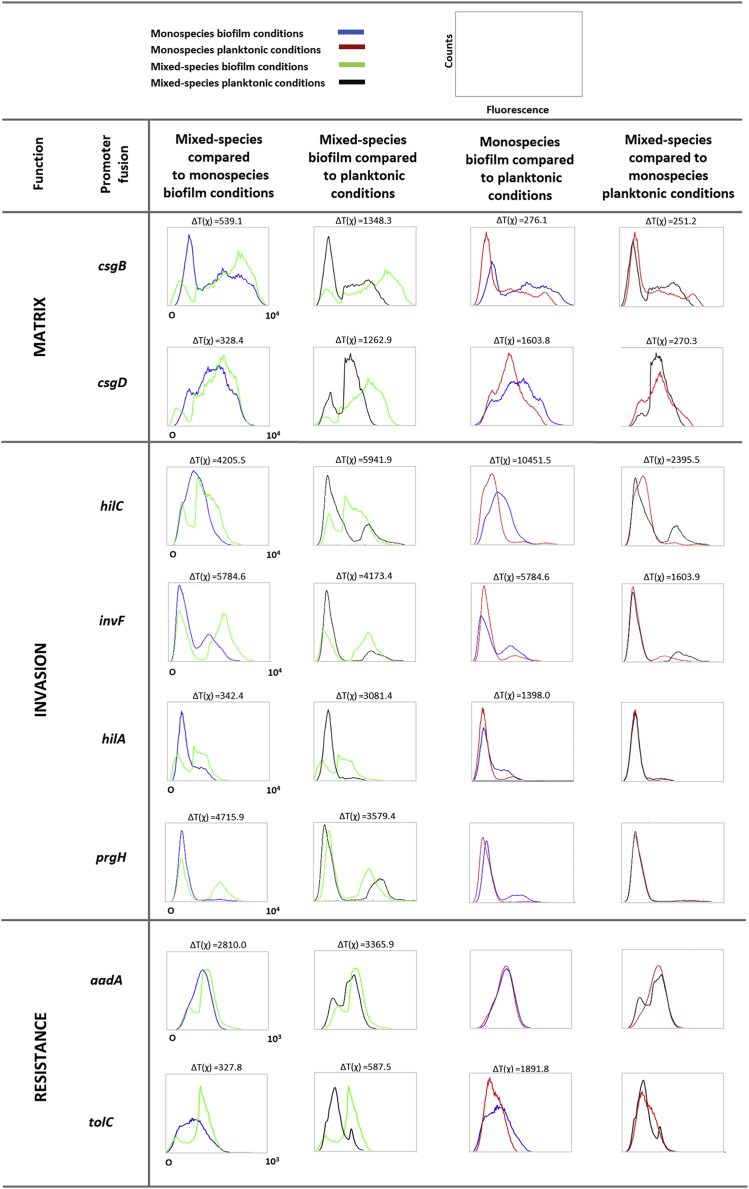
Mixed Culture Drives Up Expression of Genes Involved in Biofilm Matrix Production, Epithelial Invasion, and Antibiotic Tolerance FACS profiles of *S*. Typhimurium SL1344 (S1) genes induced by competition, with functions related to biofilm matrix production (*csgB* and *csgD*), epithelial invasion (*hilC*, *invF*, *hilA*, and *prgH*), and antibiotic resistance (*aadA* and *tolC*). Five of these genes were identified in the DFI screening (*csgB*, *hilC*, *invF*, *aadA*, and *tolC*). The other genes were selected based on knowledge of the regulatory networks. Gene expression in S1 was measured by promoter GFP fusions and FACS under four conditions: monospecies planktonic (red line); monospecies biofilm (blue line); mixed-species planktonic (black line); and mixed-species biofilm (green line). The FACS profiles show the population distribution of fluorescence in S1 under the different conditions. In each condition, 100,000 S1 cells were analyzed. Data were analyzed by using the FlowJo software and probability binning, as described in STAR Methods. For significant differences between populations (T(χ) > T(χ) _minimum_), the ΔT(χ) values are displayed. Additionally, in each pathway, the increased expression of a central regulator (*csgD*, *hilA*, and *tolC*) was confirmed using a more strict T(χ) _specific_ based on the 95% confidence interval of T(χ) of that specific reporter gene in the condition with the highest variation (n = 10). One representative repeat of at least two independent biological repeats is shown. See also Figures S2 and S3.

The five remaining genes, i.e., *csgB*, *invF*, *hilC*, *tolC*, and *aadA*, were more highly expressed in the mixed biofilm compared to the monospecies biofilm (Figure 3), indicating that the presence of competing strains is central to the induction of these genes. The *csgBAC* operon is involved in the production of curli fimbriae, one of the major components of the *Salmonella* biofilm matrix [30]. As expected, the *csgB* promoter was found to be induced in biofilm compared to planktonic state. However, a stronger induction was observed in mixed-species conditions than in monospecies conditions, and *csgB* was more highly expressed in the mixed-species biofilm compared to the monospecies biofilm. Indeed, in the mixed-species biofilm, the subpopulation with the highest expression level increases in proportion and shifts to an even higher mean expression level (Figure 3).

Expression of the SPI1 invasion genes *invF* and *hilC* was also induced in the mixed-species biofilm compared to the monospecies biofilm [31]. The SPI1 invasion system is a type III secretion system encoded on *Salmonella* pathogenicity island 1 (SPI1) and used by *Salmonella* to invade the intestinal epithelium of the host [31]. Finally, we observed upregulation of the antibiotic resistance genes *aadA* (aminoglycoside resistance) [32] and the outer membrane gene *tolC* [33]. The *aadA* gene, encoding an aminoglycoside adenyltransferase, is responsible for resistance of the cell to aminoglycoside antibiotics, such as streptomycin and spectinomycin [34]. The *tolC* gene encodes an outer membrane porin, which is a part of efflux pumps that remove diverse molecules from the cell, including antibiotics, such as quinolones, aminoglycosides, chloramphenicol, and tetracycline [35, 36].

Our selection regimen was stringent and intended to identify those genes that are strongly induced in at least a subpopulation of cells. It is, therefore, not an exhaustive method. To identify additional loci, we studied loci known to be functionally related to those discovered with DFI. Specifically, we used promoter GFP fusions and FACS under our four test conditions (mono biofilm, mixed biofilm, mono planktonic, and mixed planktonic). Consistent with the induced transcription of *csgB*, we found that its key regulator, CsgD (master regulator of matrix production in *Salmonella*), was also affected by mixed culture (Figure 3). The effect on *csgB* is most probably mediated by CsgD, as it has been reported that CsgD directly activates the expression of *csgB* [30]. Consistent with the induced transcription of *hilC* and *invF* in a subpopulation of the mixed-species biofilm compared to the monospecies biofilm, genes encoding the SPI1 regulator *hilA* and effector *prgH* were also found to be upregulated in a subpopulation of the mixed-species biofilm (Figure 3). Our observation of responses in a subpopulation of cells is consistent with previous work showing that *hilA* expression is bimodal [37]. There are a large number of potential activators of efflux pumps and antibiotic resistance loci, including several major regulators. We did not, therefore, look for specific co-regulated loci as we did for biofilm formation and invasion and instead moved to consider the general regulators associated with the responses (section below).

One concern of our method is that, rather than identifying genes that are upregulated, it might instead identify genes that allow cells to survive better in mixed-biofilm conditions. However, we excluded this possibility as deletion mutants in identified genes (*csgD*, *tolC*, and *hilA*) do not survive less well in the conditions of the assay (Figure S4B). These results also underline that our *in vitro* assay does not capture the full range of evolutionary pressures that *S*. Typhimurium experiences under natural conditions, because these major phenotypes do not carry a benefit. Understanding the evolutionary function of the responses we have identified, therefore, will likely require more complex assays, such as *in vivo* work (see Discussion).

We wanted to validate that the transcriptional responses translated to phenotypic responses. We chose this strategy rather than confirmation with other expression assays as the accumulated expression profiles measured by stable reporter fusions cannot directly be compared with the time-dependent expression profiles provided by traditional methods, such as qPCR and RNA sequencing (RNA-seq). Moreover, phenotypic assays provide the gold standard for validation, as they show that molecular changes are ultimately manifesting in relevant phenotypes at the cell and population level.

### Phenotypic Responses Recapitulate Regulatory Responses to Competition

The molecular responses to competing strains that we have discovered fit well with existing data on phenotypic responses. There are data suggesting that bacterial interactions within mixed-species biofilms can strongly enhance antibiotic resistance (or tolerance) [38, 39, 40], virulence [41, 42, 43], and biofilm formation [4, 44, 45]. However, the cause of these enhancements is unclear and, when an explanation is offered, they are typically ascribed to cooperation between species rather than being a product of competition (although see [4]). We therefore performed phenotypic assays to confirm that the presence of competing strains can indeed drive biofilm matrix formation, invasion, and antibiotic tolerance in our system.

**Figure 4:**
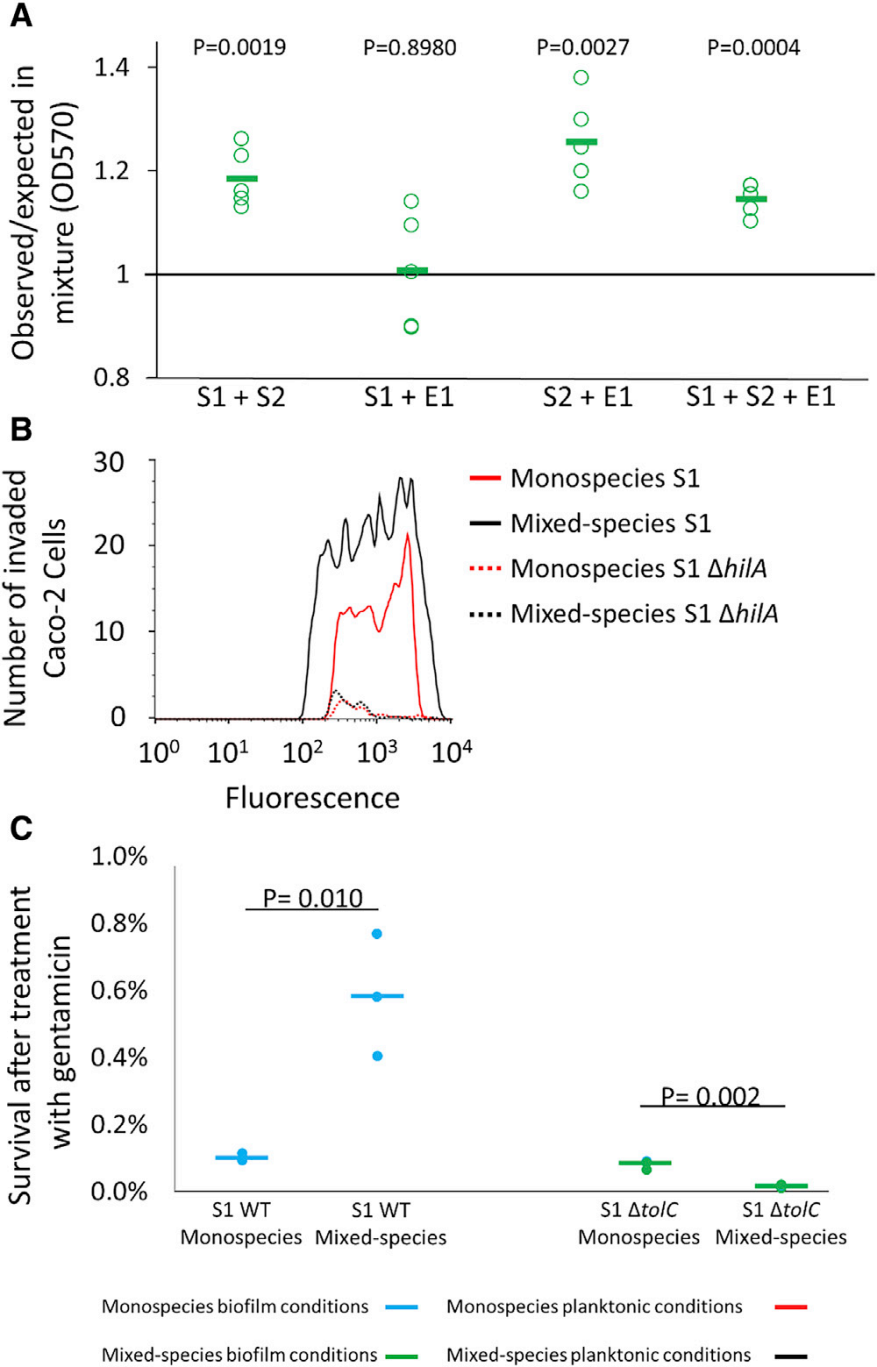
Phenotypic Assays Confirm the Regulatory Responses to Mixed Culture (A) Biofilm formation: ratio of the observed amount of biofilm (as measured by crystal violet staining) in mixed- species biofilms compared to the expected amount (STAR Methods). Biofilm production is higher than expected, confirming the biofilm response to competition. Five biological repeats and their average are shown. p values derived from one-sample t test for “greater than 1” (n = 5). (B) Invasion of Caco-2 cells: the FACS profiles show the number of invaded Caco-2 cells after exposure to fluorescently labeled S1 under monospecies (red lines) and mixed-species conditions (black lines). For each condition, the fluorescence of 10,000 Caco-2 cells was measured. The fluorescence level is determined by the number of invaded S1 cells. Only fluorescent, invaded cells are shown. Full lines represent invasion by wild-type S1 cell; dotted lines represent invasion by the isogenic Δ*hilA* mutant. A higher number of fluorescent Caco-2 cells were counted after invasion by wild-type S1 in mixed-species versus monospecies conditions, confirming that the strain interactions can trigger epithelial invasion by S1. The S1 Δ*hilA* mutant is strongly affected in invasion, confirming the need of a functional SPI1 system for Caco-2 cell invasion under the conditions tested. One repeat representative of four independent biological repeats is shown. (C) Tolerance against gentamicin: survival of S1 after 1 h incubation of pre-formed monospecies and mixed-species biofilms in the presence of 200 μM gentamicin. Survival of S1 wild-type is more than 5 times higher in mixed-species compared to monospecies conditions. This effect is abrogated in an S1 Δ*tolC* mutant. Three biological repeats and their average are shown. p values are derived from two-tailed Student’s t test using Welch’s correction if SDs are significantly (p < 0.05) different. See also Figure S4.

We first studied the effect of strain mixing on biofilm formation. As discussed above, all strains showed reduced growth in the mixed species compared to monoculture biofilms (Figure 1B). Nevertheless, it remains possible that biofilm matrix production is induced in response to competition in the mixed-species condition. The mixed-species biofilm was compared to single species biofilms by crystal violet staining. Crystal violet staining gives a proxy for the total biofilm formed, including both the extracellular biofilm matrix and the cells themselves. By comparing this total biofilm measurement to cell number, therefore, one can estimate the relative amount of matrix production in a biofilm. We compared total biofilm in mixed-species biofilms to the expected level, based on the number of cells of each strain in the mixed-species biofilm and the total biofilm per cell in each monospecies biofilm (STAR Methods). As shown in Figure 4A, the observed biofilm was generally much higher than expected in the mixed-species communities. This indicates that, as predicted by the molecular data, the average matrix production was strongly induced. This hypothesis is further supported by the observation that a mixed-species biofilm containing S1 Δ*csgD* instead of S1 wild-type no longer shows evidence of increased matrix production (Figure S4A) [16].

We next explored the functional relevance of the induction of SPI1 invasion genes by invasion experiments with Caco-2 epithelial cells. The number of S1 cells (labeled with plasmid-encoded constitutive *gfpmut3*) able to invade the Caco-2 cells was determined by flow cytometric analysis of the Caco-2 cells. A S1 Δ*hilA* mutant was found to be strongly affected in invasion, confirming the need of a functional SPI1 system for Caco-2 cell invasion under the conditions tested (Figure 4B). We compared S1 invasion in monospecies and mixed-species conditions. A higher number of Caco-2 cells were invaded by S1 in mixed-species conditions. This is in line with the higher expression of SPI1 invasion genes, confirming that competing strains can trigger host cell invasion.

Finally, we assessed the relevance of the enhanced expression of efflux pumps on antibiotic tolerance by studying the effect of gentamicin on pre-formed monospecies and mixed-species biofilms. Gentamicin is an aminoglycoside that blocks protein synthesis. Tolerance against aminoglycosides can be conferred by the AcrAD-TolC efflux pump [36]. 1 h treatment of mature (48-h-old) monospecies and mixed-species biofilms with 200 μM gentamicin resulted in a significantly higher survival of S1 grown in mixed-species conditions. Deletion of *tolC* did not affect monospecies biofilm formation (Figure S4B) but completely abrogated the enhanced tolerance of S1 in the mixed-species biofilm, confirming that the enhanced tolerance is due to increased expression of TolC efflux pumps (Figure 4C). S1 was also more tolerant in mixed-species conditions to treatment with ciprofloxacin and the bacteriostatic antibiotic tetracycline, albeit not significantly for the former. However, in both cases, the S1 Δ*tolC* mutant was not more susceptible to the treatment, suggesting that another factor, such as the increased matrix production in mixed-species conditions, may drive the enhanced tolerance of S1 (Figures S4C and S4D).

### The Response to Competing Strains Is Mediated by Stress Responses

We have shown that the presence of competing strains in our model biofilm community induces the expression of antibiotic tolerance genes, the SPI1 invasion system, and the biofilm matrix pathway. But how is *S*. Typhimurium detecting competitors? It has recently been proposed that bacteria can directly detect competition using stress responses [9]. This idea—competition sensing—came from the realization that competition, by definition, is harmful and stress responses represent ideal mechanisms for a bacterium to detect the various harms caused by competition. Moreover, previous work has shown that environmental stressors associated with competition can induce our three key phenotypes. Multiple studies have shown that sub-lethal concentrations of antibiotics can induce biofilm formation [4, 46, 47], expression of virulence genes [48, 49], and expression of antibiotic tolerance genes [50, 51] in *Salmonella* and a diverse set of other bacteria. Nutrient limitation can drive similar responses: increased biofilm formation [52, 53]; virulence [54, 55, 56]; and antibiotic tolerance [57, 58]. In many of these examples, direct links to stress response activation have been described. However, importantly, in these studies, the stress was not shown to be caused by competing strains.

**Figure 5:**
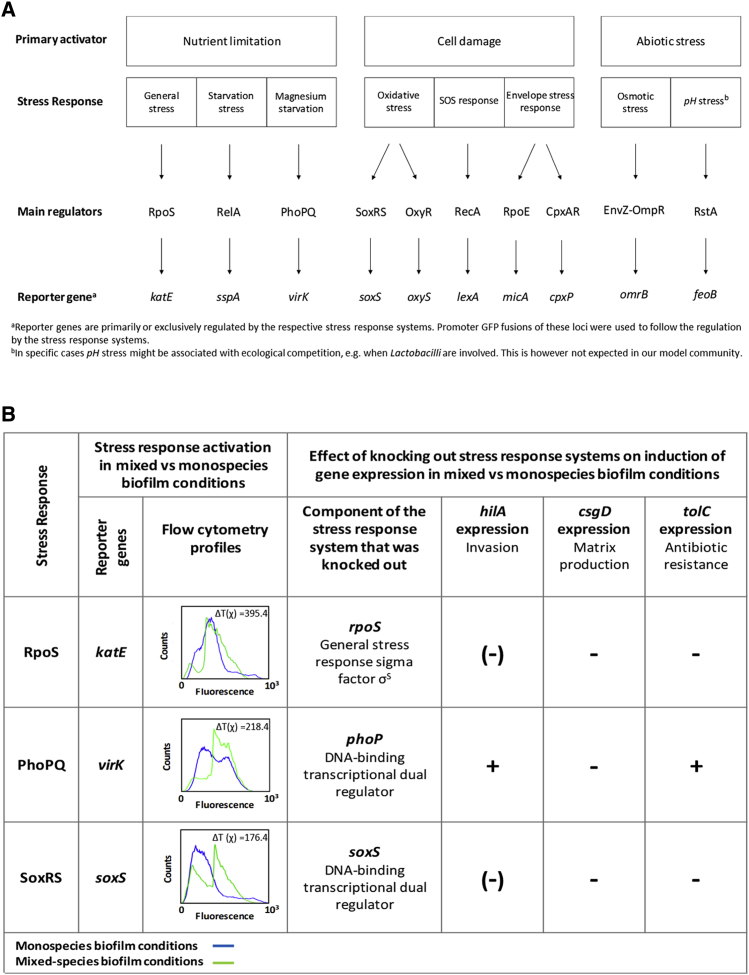
The Response to Competition Is Mediated by Stress Responses (A) Classification of stress response systems in S1 according to their primary activator. In specific cases, *pH* stress might be associated with competition, e.g., when *Lactobacilli* are involved. This is, however, not expected in our model community. Reporter genes are primarily or exclusively regulated by the respective stress response systems: *katE* [59]; *sspA* [60]; *virK* [61]; *soxS* [62]; *oxyS* [63]; *lexA* [64]; *micA* [65]; *cpxP* [66]; *omrB* [67]; and *feoB* [68]. Promoter GFP fusions of these loci were used to follow the regulation by the stress response systems. (B) Expression of reporter genes for stress response systems (left) and effect of knocking out crucial components of each of these systems on the induction of *csgD* (matrix production), *hilA* (invasion), and *tolC* (antibiotic resistance) in the mixed-species compared to monospecies biofilms (right). FACS analysis showed that reporter genes for the general stress response system mediated by RpoS (reporter gene *katE*), the PhoPQ system (reporter gene *virK*), and oxidative stress response system SoxRS (reporter gene *soxS*) were induced in mixed-species biofilm (green line) compared to monospecies biofilm (blue line) conditions (probability binning indicates that T(χ) > T(χ) _minimum_). Positive hits (*katE*, *virK*, and *soxS*) were confirmed using a more strict T(χ) _specific_ based on the 95% confidence interval of each reporter gene in the condition with the most variation (n = 10). The ΔT(χ) = T(χ) − T(χ) _specific_ is displayed. The symbols indicate the effect of knocking out the stress response systems on the induction of matrix (*csgD*), SPI1 invasion (*hilA*), and antibiotic resistance (*tolC*) in mixed versus monospecies biofilms: “−,” the induction is completely abolished in the stress response mutant; “(−),” the induction is partially abolished in the mutant, “+,” the response is still present in the mutant. One representative repeat of at least three independent biological repeats is shown. See also Figures S5 and S6.

In order to test for a direct link between mixed culture, competition, and stress responses in *S.* Typhimurium, we followed the regulation of ten major stress responses in monospecies and mixed-species biofilms. We did this with fluorescent reporters for loci that are primarily, or exclusively, regulated by each stress response system (Figure 5A). We classify these different stress response systems according to their primary activator (nutrient limitation and cell damage, e.g., by antibiotics or abiotic stress) [9].

Consistent with competition sensing, we observed that competition drove the upregulation of several stress responses associated with nutrient limitation and cell damage. Specifically, we observed upregulation of the general stress response mediated by RpoS (reporter gene *katE*), the Mg^2+^-dependent PhoPQ system (reporter gene *virK*), and oxidative stress response system SoxRS (reporter gene *soxS*; Figure 5B). Moreover, this induction was abolished when crucial components of the respective stress response systems were knocked out, which confirms that each reporter gene does indeed report on its respective stress response (Figure S5A).

We then determined whether the induction of *csgD* (matrix production), *hilA* (invasion), and *tolC* (efflux) in the mixed-species biofilm is linked to the observed stress responses. We measured the expression of each of the three loci in the respective stress response knockouts in the four conditions (monospecies biofilm, monospecies planktonic, mixed-species biofilm, and mixed-species planktonic). The induction of all three genes in the mixed-species compared to monospecies biofilm was largely abrogated when the general stress response (Δ*rpoS*) or oxidative stress response (Δ*soxS*) was knocked out (a subpopulation of the *soxS* mutant showed a non-significant increase in *hilA* expression). Inactivation of the PhoPQ system (Δ*phoP*) only abolished the induction of *csgD* (Figures 5B and S6).

Deleting stress response regulators can have large effects on cell physiology and leads to the potential for pleiotropic effects that alter downstream phenotypes in a non-specific way. To control for such effects, we artificially activated two of the stress response systems using known triggers, where stress responses were again monitored via fluorescent reporters (Figure S5B). The effect of inducing RpoS was not studied due to a lack of RpoS-specific inducers. For the other two, we found that paraquat, a known trigger of the SoxRS system [62], induced the expression of *hilA* and *tolC* in monospecies conditions, and C18G, a trigger of the PhoPQ system [69], upregulated the expression of *csgD* (Figure S5B).

### T6SS-Mediated Competition Induces the Responses to Competition

Both deletion and artificial activation of stress responses support a causal link to the upregulation of competition-induced phenotypes. But what are the stress responses responding to in mixed culture? Multiple triggers are possible. Each system is known to respond to a diversity of stresses, including nutrient starvation [70]; low pH [71]; and diverse forms of cell damage, including oxidative stress [72], bacterial toxins, phages, and antibiotics [73, 74]. We hypothesized that interference competition was likely to play a significant role in the responses due to the negative complementarity between the strains (Figure 1C), and unlike nutrient limitation, cell damage is known to activate all of RpoS, SoxRS, and PhoPQ.

We first determined whether the trigger of the stress responses in S1 in mixed culture is a secreted substance or whether the physical presence of the other species is needed. Cell-free supernatant experiments indicated that factors secreted by competitors or nutrient limitation do not induce the response to competition (Figure S7A), suggesting that contact-dependent mechanisms are involved. The best studied and arguably the most important system responsible for contact-dependent competition is the T6SS. The T6SS of *S*. Typhimurium is encoded within *Salmonella* pathogenicity island 6 (SPI6) and is essential for the establishment of *Salmonella* in the gut via the injection of Tae4 effector proteins that cleave the peptidoglycan scaffold of competing gram negatives [75]. Because the E1 strain does not encode an active T6SS [76], we focused on the competition between the two *Salmonella* strains and constructed a S2 deletion mutant lacking ClpV, an ATPase essential for T6SS function [75], hereafter referred to as S2 ΔT6SS. This mutant was unaffected in biofilm formation (Figure S7B).

**Figure 6:**
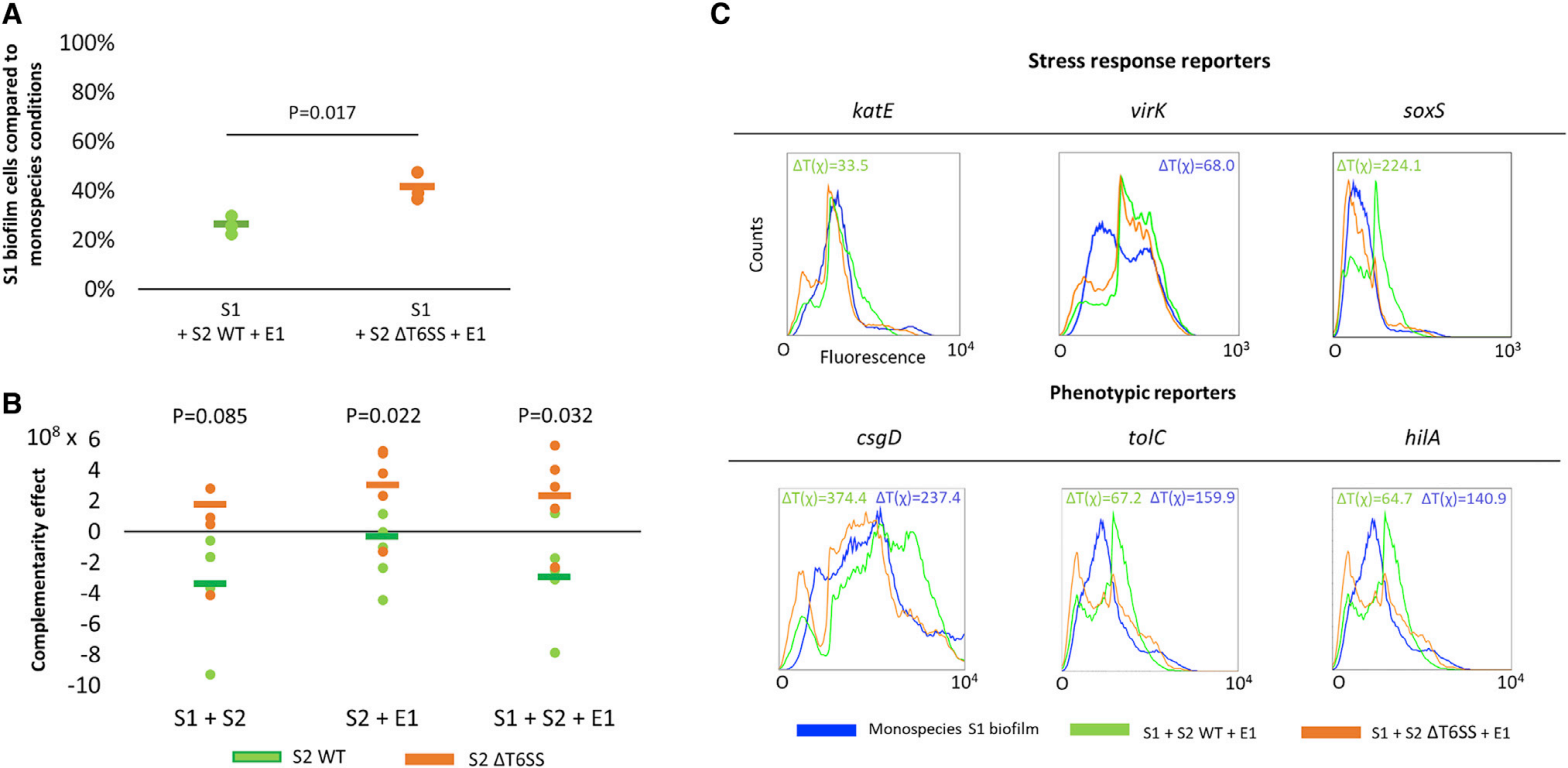
Inactivation of the T6SS in S2 Significantly Reduces the Inhibition of S1, the Level of Total Interference Competition, and the Competitive Response of S1 (A) The biofilm cell counts of S1 in the presence of a community containing either the S2 wild-type or the S2 ΔT6SS deletion mutant. S1 is inhibited to a lower extent by the presence of the other strains if the T6SS of S2 is inactivated. p values are derived from two-tailed Student’s t test using Welch’s correction if SDs are significantly (p < 0.05) different. (B) The complementarity effect of mixed-species cultures. The mixed-species biofilm model, as well as the pairwise combination of S1 and S2, no longer show negative complementarity when the T6SS of S2 is inactivated. Five different biological repeats and their average are shown. p values are derived from two-tailed Student’s t test using Welch’s correction if SDs are significantly (p < 0.05) different. (C) The expression of stress response reporters *katE*, *virK*, and *soxS* and phenotypic reporters *csgD* (matrix production), *hilA* (virulence), and *tolC* (antibiotic resistance) in S1 when grown in monospecies conditions (blue) and in the presence of competitors with (green) and without functional T6SS (orange). The FACS profiles show the population distribution of fluorescence in S1 under the different conditions. In each condition, 100,000 S1 cells were analyzed. Data were analyzed by using the FlowJo software and probability binning. Significant differences (T(χ) > T(χ) _specific_) between mixed-species populations containing S2 wild-type (WT) and S2 ΔT6SS are indicated with a green ΔT value; significant differences (T(χ) > T(χ) _specific_) between mixed-species populations containing S2 ΔT6SS and monospecies populations of S1 WT are indicated with a blue ΔT value. The T(χ) _specific_ is adapted for each specific reporter gene based on the 95% confidence interval of T(χ) of that reporter gene in the condition with the highest variation (n = 10). One representative repeat of at least two independent biological repeats is shown. See also Figures S6 and S7.

Inactivating the T6SS in S2 significantly increased the cell number of S1 in mixed-species conditions (Figure 6A), indicating that a T6SS-mediated attack by S2 indeed inhibits S1. We also studied the level of interference competition between the *Salmonella* strains by calculating the complementarity effect [25] (Figure 6B). The complementarity levels of the biofilms containing S1 and the S2 ΔT6SS mutant are no longer negative, consistent with the T6SS of S2 driving interference competition between the *Salmonella* strains. When inoculating S1 together with S2 ΔT6SS and E1, activation of the stress response regulators RpoS and SoxRS (but not PhoPQ) was lower than in the wild-type community, but not completely abrogated (Figure 6C). The T6SS, therefore, does partly explain the activation of the stress responses. However, in the absence of the T6SS in strain S2, the expression of *csgD*, *tolC*, and *hilA* was no longer upregulated in S1. Stress caused by the T6SS, therefore, is sufficient to explain all of the downstream responses to competition.

In sum, these results indicate that the cell damage caused by T6SS-mediated competition exerted by S2 is detected by the RpoS and SoxRS stress response systems of S1, leading to increased biofilm formation, invasion, and antibiotic tolerance. Consistent with the strong effect of inactivating the T6SS of S2, we also found that E1 alone could not induce any response in S1, further confirming that it is S2 that triggers the responses in S1 (Figure S7C).

## Discussion

We have discovered a diverse and strong set of molecular responses to competing strains, including enhanced expression of efflux pumps (*tolC*), invasion (*hilA*), and matrix production (*csgD*) genes. These molecular responses add to a growing body of phenotypic data showing that major bacterial traits are affected by competition or cell damage, including biofilm formation, siderophore production, antibiotic production, and efflux pump upregulation [4, 9, 10, 11, 77, 78]. In addition, our work provides a critical missing link by showing that major stress response systems, including those driven by RpoS, SoxRS, and PhoPQ, are responsible for detecting and responding to competition in biofilms. Overall, these findings suggest that competition might explain the previously reported increase in biofilm [44, 45], tolerance against antimicrobials [38, 39, 40], and virulence [41, 43] in mixed-species communities for which competitive interactions were not yet fully characterized.

We found fewer genes upregulated in mixed-species conditions than studies investigating transcriptional responses to competition [79]. The limited number of positive hits is likely explained by our stringent experimental design that only selects for genes that show an increased accumulated expression over a 48-h period, across multiple rounds of selection [14]. In contrast, traditional methods, such as RNA-seq or qPCR, measure real-time expression and would also identify genes that have a modified timing of expression in mixed-species conditions due to altered growth. There may, therefore, be other responses that we did not pick up with our method. Moreover, we screened specifically for genes that are associated with strong competition (= mixed-species biofilm) and discarded genes that are already induced in the presence of another strain (= mixed-species planktonic), whereas other studies directly compare expression between mono- and mixed-species conditions. However, the stringency of our method is also a strength in that we were able to identify molecular responses that were all validated in concomitant changes in phenotype. Moreover, although relatively few genes were implicated by the screen, they led to the discovery of changes associated with three of the major bacterial phenotypes (biofilm, invasion, and drug resistance) and associated stress response regulators.

Remarkably, a significant proportion of the identified genes show a bimodal expression pattern, even though the experimental setup of our screening removed genes selected solely because of their noisy or bimodal expression. One possible explanation is that bimodality allows bacteria to respond quickly to competitors. Due to the bimodality in a pathway that protects against competition, a part of the population would already be protected against an attack without the need to first change expression, similar to a bet-hedging strategy [80]. Another potential hypothesis is that most social behavior is inherently public, as it requires directly influencing the fitness of other bacteria. A bimodal expression pattern could stabilize publicly beneficial social behavior and protect against exploitation by cheaters, as the cheaters do not have a significant fitness benefit compared to the subpopulation with (almost) no expression [81].

Our data indicate that *S*. Typhimurium is performing competition sensing and using stress responses to detect competition [9]. Specifically, we found that the induced expression of biofilm matrix, epithelial invasion, and efflux is abrogated in mutants of the stress response systems meditated by RpoS, SoxRS, and PhoPQ. A potential limitation of studying stress responses is that deletion of these systems can have pleiotropic effects on the growth and metabolic activity of the cell, possibly causing systematic perturbations in gene expression not specific to the responses to competition. However, we also found that specific activation of the stress response systems in monospecies conditions elicits responses that are similar to the responses to competition, which allays the concern associated with deletion mutants (Figure S5B). In line with these results, previous work has shown that treating *Salmonella* with triclosan activates both the SoxRS and RpoS stress response systems and induces the expression of *csgD*, *tolC*, and SPI-I genes [82]. Additionally, activation of the PhoPQ system via Mg^2+^ limitation has been associated with increased expression of *csgD* and *tolC* [83]. Moreover, molecular links between RpoS and PhoPQ and the *csgD* and SPI1 pathways have been described [30, 84, 85], and it has been reported that *tolC* expression is mediated by PhoPQ and SoxS [86, 87]. However, no direct regulatory links have been described between SoxRS and the *csgD* and SPI1 pathways or between RpoS and *tolC*. The lack of direct molecular link described in literature could be a consequence of the stress response systems traditionally being studied using a single stressor in contrast to the complex stress provided by the competing strains in our study.

We also demonstrated that T6SS-dependent competition is an important trigger of RpoS and SoxRS, whereas the trigger of PhoPQ remains unknown. This trigger is likely not a secreted compound, such as quorum-sensing molecules or bacteriocins, as PhoPQ was not activated in our cell-free supernatant assay. Given that S2 has low expression of pili fimbriae [88] and no contact-dependent inhibition (CDI) systems have been described, it may be that PhoPQ is activated due to interspecies resource competition in our model community. The fact that T6SS does not explain all of the stress response activation, while being sufficient to fully induce the downstream responses, suggests that strain S1 is using multiple redundant mechanisms to detect S2. Such redundancy in information gathering is well known from diverse biological systems and is expected to evolve whenever errors are costly, such that a correct assessment is important for fitness [89].

What is evolutionary function of the observed responses to competition? We hypothesize that the responses allow strains to better cope with the stress caused by other strains under natural conditions. This is consistent with what is known about biofilm formation and efflux pumps, which can both protect bacteria against harm. For example, growth in biofilms commonly leads to large clonal patches that can inhibit the ability of one strain to inhibit the other via the T6SS [3, 90]. However, one should not expect the responses to only provide an advantage in the face of a T6SS attack. Stress responses activation can be used by cells as predictor of a range of threats that come with competition [9]. Consistently, biofilm matrix components, including cellulose and curli fimbriae, are well known to promote tolerance to a wide range of stressors, such as antibiotics and toxins from competing strains [91, 92]. In addition, the biofilm matrix has been shown to increase nutrient availability, either by adsorption of nutrients [91, 93] or by providing better access to nutrients [93], thereby likely playing a role in exploitation competition. Furthermore, recent reports indicated that the biofilm matrix can provide protection against invasion by competitors and thus exclude competing strains [94].

The increase in antibiotic tolerance that we observe is arguably the most understandable response in terms of bacterial competition. Enhanced expression of antibiotic resistance mechanisms, such as efflux pumps and aminoglycoside-modifying enzymes, are likely to provide protection against a range of toxins secreted and injected by competing bacteria [35, 36]. Combined with our findings, these data suggest that the root of the commonly reported antibiotic tolerance of microbial consortia may lie in the response of bacteria to competition between strains, not in cooperation, as has been suggested [5, 44]. This realization has implications for how we treat and manage microbial communities. One emerging strategy is the use of probiotic species, or whole communities in the case of fecal transplants, that compete for resources with pathogens or produce antimicrobial compounds [95], something that our data suggest can increase antibiotic tolerance. If so, an interesting alternative would be to specifically inhibit traits [96] that allow a strain to compete, such as T6SS or antibiotic production. This strategy has the potential to both reduce the frequency of a focal strain *and* competition-associated traits in the remaining community.

Less obvious is the invasion response, which may instead be a byproduct of an evolved response to host-derived stress during infection that is triggered by the T6SS in our experiments. Such misactivation is plausible, as stress responses commonly respond to a broad range of stressors, e.g., *soxS* induction by either T6SS, phage, or antibiotics [80]. This said, the co-regulation with biofilm formation and efflux may also indicate a common basis for the responses. Consistent with this, the establishment of *Salmonella* in the gut is intimately tied to its ability to compete with the resident intestinal microbiota [97, 98]. Invasion of the gut tissue by a subpopulation of the *Salmonella* bacteria triggers an inflammatory response that helps *Salmonella* to compete with the resident microbiota through several mechanisms [55, 97, 98], including the generation of tetrathionate used by *Salmonella* [98] and the epithelial release of specific antimicrobials to which *Salmonella* is resistant [99]. Moreover, the intensity of inflammation increases as the proportion of bacteria that are capable of invading increases [100]. Given this, *Salmonella* may benefit from increasing epithelial invasion because this amplifies the impacts of inflammation on its competitors.

The complex regulatory networks bacteria use to cope with environmental challenges have been brought into new focus by the imminent threat of antibiotic resistance. Our work suggests that understanding and predicting these responses requires attention to how bacteria typically live: embedded in dense, diverse, and competitive communities. These are the conditions that bacteria faced for billions of years prior to the clinical use of antibiotics, conditions that they continue to face today.

## STAR★Methods

### Key Resources Table

**Table:** 

REAGENT or RESOURCE	SOURCE	IDENTIFIER
**Bacterial and Virus Strains**		

*Salmonella* Typhimurium SL1344	[15]	SL 1344
*Salmonella* Typhimurium SL1344 Δ*rpoS*	[101]	N/A
*Salmonella* Typhimurium SL1344 Δ*phoP*	This paper	N/A
*Salmonella* Typhimurium SL1344 Δ*soxS*	This paper	N/A
*Salmonella* Typhimurium SL1344 Δ*soxR*	This paper	N/A
*Salmonella* Typhimurium SL1344 Δ*hilA*	[102]	N/A
*Salmonella* Typhimurium SL1344 Δ*csgD*	[16]	N/A
*Salmonella* Typhimurium SL1344 Δ*tolC*	This paper	N/A
*Salmonella* Typhimurium ATCC14028	ATCC	ATCC:14028
*Salmonella* Typhimurium ATCC14028 Δ*clpV*	This paper	N/A
*Escherichia coli* MG1655	ATCC	ATCC:47076

**Chemicals, Peptides, and Recombinant Proteins**		

Ampicillin sodium salt	Sigma	Cat# A9518; CAS:69-52-3
Streptomycin sulfate salt	Sigma	Cat# S6501; CAS:3810-74-0
Paraquat dichloride hydrate	Sigma	Cat# A36541; CAS:75365-73-0
Chloramphenicol	Sigma	Cat# C1200000; CAS:56-75-7
Human Platelet Factor IV 18, C18G	AnaSpec	Cat# AS-6241

**Deposited Data**		

Raw sequence of positive hits DFI screening	This paper; Mendeley Data	https://doi.org/10.17632/vst2c592mt.2

**Experimental Models: Cell Lines**		

Caco-2 cell line	ATCC	ATCC:HTB-37; RRID: CVCL_0025

**Oligonucleotides**		

Primers - see Table S1		

**Recombinant DNA**		

Plasmids – see Table S2		

**Software and Algorithms**		

Graphpad Prism 6	GraphPad Software	https://www.graphpad.com
MATLAB 9.2	MathWorks	https://www.mathworks.com/
Flowjo 7.6.5	Becton, Dickinson and Company	https://www.flowjo.com/
Zen Blue 2011	Zeiss	https://www.zeiss.com

### Lead Contact and Materials Availability

Further information and requests for resources and reagents should be directed to and will be fulfilled by the Lead Contact, Hans Steenackers (hans.steenackers@kuleuven.be). Genomic mutants and plasmids constructed for this study can be made available upon request following the signing of a material transfer agreement.

### Experimental Model and Subject Details

#### Growth conditions

*Salmonella* Typhimurium SL1344 (S1), *S.* Typhimurium ATCC14028 (S2) and *Escherichia coli* (E1) wild-type strains were grown overnight in lysogeny broth (LB) at 37°C with continuous shaking at 200 rpm or on solid LB agar plates (15 g/l, Invitrogen). For biofilm growth conditions, the overnight cultures of S1, S2 and/or E1 were diluted 1:100 in Petri dishes containing 10 mL Tryptic soy broth (TSB) 1/20 (1.5 g/l, BD Biosciences) with ampicillin (Ap, 100 μg/ml) and biofilms were grown statically for 48 h at 25°C on the bottom of the dishes (60 mm diameter, Greiner Bio-One). The same total number of cells was inoculated in mono- and mixed-species conditions. Only in the assays that determine the type of interaction (Figures 1B, S1A, and S1B), the number of cells for each species was the same in in mono- and mixed-species conditions and thus three times as many cells were inoculated in mixed-species conditions (as described in [8]). After incubation, the liquid above the biofilms was poured off and the biofilms were scraped off the bottom of the plate in 1 mL of FACSFlowTM Sheath Fluid (Becton Dickinson), passed through a syringe (25G) and vortexed to break down the biofilm structure and avoid clumps during subsequent DFI analysis as described earlier [16]. For planktonic growth, overnight cultures of S1, S2 and/or E1 were diluted 1:100 in test tubes containing 5 mL TSB 1/20 broth with Ap antibiotics and grown overnight with aeration (200 rpm) at 25°C.

#### Plasmids and mutants

The pFPV25.1 plasmid expressing *gfp*mut3 in a constitutive fashion from an *rpsM* promoter was kindly provided by Raphael H. Valdivia and Stanley Falkow and renamed pFPV25.1_GREEN. To construct a similar plasmid with constitutively expressed *dsred.T4*, the *dsred.T4* gene was PCR amplified. The *gfp*mut3 gene was removed from pFPV25.1_GREEN by restriction digestion and the PCR amplified *dsred.T4* was subsequently cloned into the same region. Restriction enzymes were purchased from Roche and used according to the instructions of the manufacturer. *E. coli* DH5α and *E. coli* Top10F’ were used for cloning steps. The new construct was confirmed by PCR amplification, sequence analysis and named pFPV25.1_RED. *S.* Typhimurium strains were provided with the appropriate plasmids by electroporation (Bio-Rad gene pulser). Knock-out mutants in S1 and S2 were constructed via the one-step chromosomal inactivation protocol according to Datsenko and Wanner [103]. Spefically, the gene of interest was replaced with an antibiotic resistance cassette flanked by FLP recognition target sites via homologs recombination using the phage lambda Red recombinase encoded on the curable pKD46 plasmid. Homologs regions of 35 to 50 bp were generated via primer extension. Successful knock-out mutants were selected via plating on agar plates containing chloramphenicol. Afterward, resistance cassettes were removed via an FLP recombinase encoded on a curable pCP20 helper plasmid in order to avoid unwanted side-effects. All strains and constructs were verified by PCR and sequencing analysis. All primers and plasmids used for the construction of these knock-out mutants are listed respectively in Tables S1 and S2.

#### Promoter-probe library construction of S. Typhimurium SL1344

A full description of the library construction was published before [16]. Briefly, the genomic DNA of *S*. Typhimurium SL1344 was partially digested with the Sau3AI restriction enzyme. The digest was size-fractionated via agarose gel electrophoresis. DNA fragments of 0.4–1.6 kb were selected and inserted into the BamHI site upstream of a promoterless *gfpmut3* gene in pFPV25. This *gfpmut3* gene encodes a highly stable GFP variant with a half-life time longer than 24 h [14, 104]. The obtained plasmid library was electroporated into wild-type S1 cells, yielding approximately 20500 clones.

### Method Details

#### Complementarity effect

Biofilms were incubated as described in the section ‘growth conditions’. For these experiments, the same total number of cells was inoculated in mono- and mixed-species conditions. The complementarity effect was calculated according to the formula below. This effect measures whether the relative amount of cells in mixed-species conditions is on average higher or lower than expected based on the initial relative abundance and growth in monospecies conditions [12].

$$\text{Complementarity}=N\bar{\Delta RY}\bar{M}$$

N = number of species in mixed-species community$M_{i}=$ growth of species i in monospecies conditions$RY_{E,i}=$ expected relative biofilm growth of species i in mixed-species conditions, which is its proportion inoculated$RY_{O,i}=Y_{O,i}/M_{i}=$ observed relative growth of species i in mixed-species$\Delta RY_{i}=RY_{O,i}-RY_{E,i}$ = deviation from expected relative growth of species i in mixed-species conditions

#### Flow cytometric analysis

Analysis of bacterial cell suspensions was done by using a BDInflux (Becton Dickinson). Bacteria harboring pFPV25 and pFPV25.1 were used as negative and positive controls respectively to optimize the instrumental settings to our needs. Fluorescence, forward and side scatter data were collected for 10^5^ cells to distinguish between debris and cells. Prior to each analysis a calibration was performed using SPHEROTM Rainbow Calibration Particles, 8 peaks, 3.0-3.4 μm (Spherotech), according to the manufacturer’s recommendations.

#### DFI enrichment and individual profiling of biofilm-induced promoter fusions

The constructed promoter-probe library was divided in 21 random pools of approximately 1000 clones. In a first positive selection round, each pool was subjected to mixed-species biofilm growth conditions in co-culture with S2 and E1 containing the pFPV25.1_RED plasmid which bears a constitutively expressed *dsred.T4* gene. After 48h, 10^5^ of the harvested biofilm cells were analyzed by fluorescence-activated cell sorting (FACS), sorting green fluorescent bacteria exceeding a pre-determined threshold fluorescence value (at single cell mode [26]). This threshold was pre-defined by FACS analysis of a biofilm containing the whole promoter-probe library, and set at the level of the 5% most fluorescent clones. To exclude genes that are not associated with competition from the subpool of sorted cells, this subpool was subsequently amplified by overnight growth in LB and subjected to mixed-species planktonic conditions. In this negative selection round only non-fluorescent cells were sorted and enriched. A second positive selection round resulted in a final subpool of S1 cells with plasmids containing promotor DNA fragments specifically induced in the mixed-species biofilm compared to the mixed-species planktonic conditions

After the final positive selection round, each collected pool was grown overnight in LB with Ap and Sm and subsequently plated on LB agar plates containing Ap and Sm. 96 colonies of each pool were individually profiled for their fluorescence expression in mixed-species biofilm and planktonic growth conditions, with the same instrument settings as optimized for the sorting. The fluorescence distribution of the clones was determined by making graphical overlays of planktonic compared to biofilm growth conditions (FlowJo software version 7.6.5). A correction factor was calculated to exclude background species and dead cells from the non-fluorescent subpopulation (for details see Quantification and Statistical Analysis). For each clone, the FACS profiles of biofilm and planktonic conditions were compared by probability binning to exclude false positives (see ‘Quantification and Statistical Analysis’). Also, a cluster analysis was performed to group different clones with similar FACS profiles, as there is a high probability that the same promoter region is generating these similar profiles. The plasmid DNA upstream of *gfpmut3* of two to twenty clones per cluster (depending on the cluster size) was sequenced to identify the promoters upregulated under mixed-species biofilm conditions. The DNA sequence in the promoter-probe plasmid was determined using primers PRO4 and PRO0406 (Sanger ABI 3730 xl; GATC Biotech) and the sequences were compared to the complete genome sequence of S1 [105] by making use of the BLASTn algorithm [106]. In each case, the promoters identified per cluster were identical, confirming the validity of our approach. However, because the promoter-trap library was built with random S1 genomic DNA fragments, one cannot exclude other effects during library construction that might influence the DFI analysis. Defined promoter-*gfpmut3* fusions were constructed for the identified promoters and profiled under monospecies and mixed-species biofilm and planktonic conditions.

#### Matrix production

Overnight cultures of S1, S2 and E1 were diluted 1:100 in 10 mL TSB 1/20 with appropriate antibiotics and grown for 48 h at 25°C on the bottom of a Petri, as previously described, in both monospecies and mixes-species conditions. Total biofilm was determined with crystal violet staining as previously described [16]. Biofilms were washed with 5 mL phosphate buffered saline (PBS), stained with 5 mL crystal violet solution, washed with 5 mL demineralized water and destained with 5 mL acetic acid (30%). Optical density at 570 nm of 200 μl resolubilized stain was determined as measure for the amount of biomass.

The expected matrix production in the mixed-species community is based on the matrix production per cell in monospecies conditions and the number of cells of each strain in the mixed-species conditions:

$$Expected\;matrix=\sum_{all\;strains}\frac{matrix\left(OD\;570\right)}{\text{cells}\;in\;monoculture\left(CFU\right)}*\left(n\;cells\;in\;mixed\;culture\left(CFU\right)\right)$$

#### Antibiotic tolerance test

Gentamicin and ciprofloxacin: monospecies and mixed-species biofilms were grown on Petri dishes for 48 h at 25°C, as described in the section ‘Growth conditions’. The medium was then replaced by 10 mL PBS with 200 μM gentamicin or 1 μM ciprofloxacin and the biofilms were incubated for an additional 1h.

Tetracycline: monospecies and mixed-species biofilms were grown on Petri dishes for 48 h at 25°C, as described in the section ‘growth conditions’. The medium was then replaced by 10 mL fresh TSB 1/20 with 75 μg/ml tetracycline and the biofilms were incubated for an additional 24 h.

Subsequently the biofilms were scraped off and plated out on solid LB agar plates for CFU determination. To differentiate between the strains, S1 was labeled with constitutive *gfpmut3* on a plasmid, while S2 and E1 were labeled with plasmid-encoded constitutive *dsRed.T4*. Differences in colony shape and size allowed differentiation between S2 and E1 during CFU counting.

#### Invasion of Caco-2 cells

Prior to the invasion tests, Caco-2 cells were allowed to adhere and grow in 12-well plates in 1,5 mL Dulbecco’s Modified Eagle Medium/Ham’s F-12 medium (DMEM-F12) without Fetal Bovine Serum (FBS) for 12 days at 37°C in a CO_2_ incubator. The growth medium was refreshed every 3 days. Afterward, Caco-2 cells were washed twice with pre-warmed (37°C) PBS. Bacterial cells were subsequently added and allowed to invade the Caco-2 cells for 2 h at 37°C in the CO_2_ incubator. The invading bacteria either consisted of S1 (pFPV25.1_GREEN) (monospecies condition) alone or an equal mixture of 10^7^ cells in total of S1 (pFPV25.1_GREEN, S2 (pFPV25.1_RED) and E1 (pFPV25.1_RED) (mixed-species condition). The total number of S1 cells in both conditions was kept equal in order to be able to compare the number of invaded S1 cells. After infection, the Caco-2 cells were treated with gentamycin to kill extra-cellular bacteria. Subsequently, the Caco-2 cells were washed twice with a mixture of pre-warmed (37°C) PBS and 100μl trypsin-EDTA 1x (Life technologies) for 10 min at 37°C and finally re-suspended in 900μl PBS. The green fluorescence of the invaded Caco-2 samples was analyzed by FACS for determination of the total invaded S1 cells (labeled in green) under both monospecies and mixed-species conditions. Data analysis was performed with the FlowJo software.

#### Cell-free supernatant assay

The supernatant of 24h old mono- and mixed species biofilms was isolated, spun down (3000 g, 10’), and filtrated using 0.025 μM filters (Millipore). The cell-free supernatant assay used the same methods for biofilm growth as described in the in the section ‘Growth conditions’, except that the S1 strain was inoculated in media containing 30% cell-free supernatant.

### Quantification and Statistical Analysis

All data shown here were collected from at least 3 parallel biological cultures (n). Data were analyzed either by unpaired Student’s t test using Welch’s correction if s.d. are significantly (p < 0.05) different or by one sample t test. In case of multiple comparisons, one-way ANOVA with Bonferroni correction was employed.

#### Probability binning

Simple comparison of population means of gene expression does not fully take into account the gene expression distribution within the population. Therefore probability binning [27], a non-parametric technique related to the Cox Chi-square approach, was used to compare FACS profiles. Probability binning selects bins such that each bin of the control sample contains the same number of events and subsequently applies these bins to the test sample. The number of events in each corresponding bin is then compared between test and control sample by using a Cox Chi-square test. The Chi Squared value is next conversed to a normalized T(χ) metric that is analogs to a t-score and describes the similarity between two distributions, independent of the number of events or bins. To determine the minimum value of T(χ) indicating a biologically significant difference between populations (i.e., the baseline T(χ): T(χ) _minimum_), wild-type S1 cells containing pFPV25.1_GREEN (constitutively expressing *gfpmut3* from an *rpsM* promoter) were grown both in mixed species biofilm and planktonic conditions (a total of 10 repeats). After FACS analysis, the probability binning algorithm was repeatedly applied to compare and determine T(χ) for the obtained biofilm and planktonic FACS profiles (n = 10). T(χ) _minimum_ was next determined as the upper limit of the 95% confidence interval of the mean of T(χ). The resulting baseline T(χ) was used as a threshold when comparing FACS profiles to exclude population differences related to impreciseness of the experimental/analytical platform, random gene expression variation or copy number variation. Prior to statistical analysis, the FACS profiles were corrected to remove a residual population of non-fluorescent cells, as detailed in ‘Correction of FACS profiles for background species and dead cells’. More stringent baseline T(χ)_specific_ values were determined for a limited set of specific focal genes, i.e., *csgD*, *tolC*, *hilA*, *katE*, *virK*, and *soxS*, by repeatedly measuring their FACS profiles under the 4 test conditions (mono- and mixed-species, plankton and biofilm) (n = 10). For each gene T(χ) was calculated repeatedly under each condition by comparing the obtained FACS profiles. T(χ) _specific_ was determined for each gene as the upper limit of the 95% confidence interval of the mean of T(χ) in the condition with the highest variation (Figure S2B).

#### Correction of FACS profiles for background species and dead cells

Flow cytometric analysis of S1 containing pFPV25.1_GREEN (constitutive GFP expression) under mixed species conditions revealed a residual population of non-fluorescent cells. This is an artifact of the technique, either caused by loss of plasmid by the cells, low expression of dsRed by a fraction of the S2 and E1 cells or dead cells present in the sample. Since this artifact could cause an underestimation of the green fluorescent signal of the reporter fusions, a correction factor for both biofilm and planktonic samples was determined. Hereto, 10 000 cells from the small non-fluorescent subpopulation were isolated by FACS and subsequently plated out for cell counting. In total, 10 individual samples were tested for both planktonic and biofilm conditions. In this way, it was possible to determine total viable cell count on the plates, as well as the fractions of the different strains. In planktonic conditions, an average of 76% were dead cells, with 59% of the remaining living cells being S1, i.e. green cells, and 41% being red cells. In biofilm conditions around 63% of the cells were dead, with a higher level of living cells being red (65%) compared to green (35%). Deletion of *csgD*, *tolC*, *hilA*, *rpoS*, *phoP*, or *soxS* in S1 did not significantly alter the composition of this non-fluorescent sub-population. Based on this information a mean correction factor to exclude background species and dead cells from this non-fluorescent subpopulation was calculated.

### Data and Code Availability

The datasets generated during this study are available at Mendeley Data at https://doi.org/10.17632/vst2c592mt.2

## Supplemental Information

Document S1. Figures S1–S7 and Tables S1 and S2

Document S2. Article plus Supplemental Information
