# Supplementary material for: Biofilm Bacteria Use Stress Responses to Detect and Respond to Competitors
Source: Curr Biol. 2020 Apr 6;30(7):1231–1244.e4. doi: 10.1016/j.cub.2020.01.065 (PMC7322538; doi:10.1016/j.cub.2020.01.065)
Supplement: Document S1. Figures S1–S7 and Tables S1 and S2 [file mmc1.pdf]

Current Biology, Volume *30*

Supplemental Information

Biofilm Bacteria Use Stress Responses to Detect and Respond to Competitors

Bram Lories, Stefanie Roberfroid, Lise Dieltjens, David De Coster, Kevin R. Foster, and Hans P. Steenackers

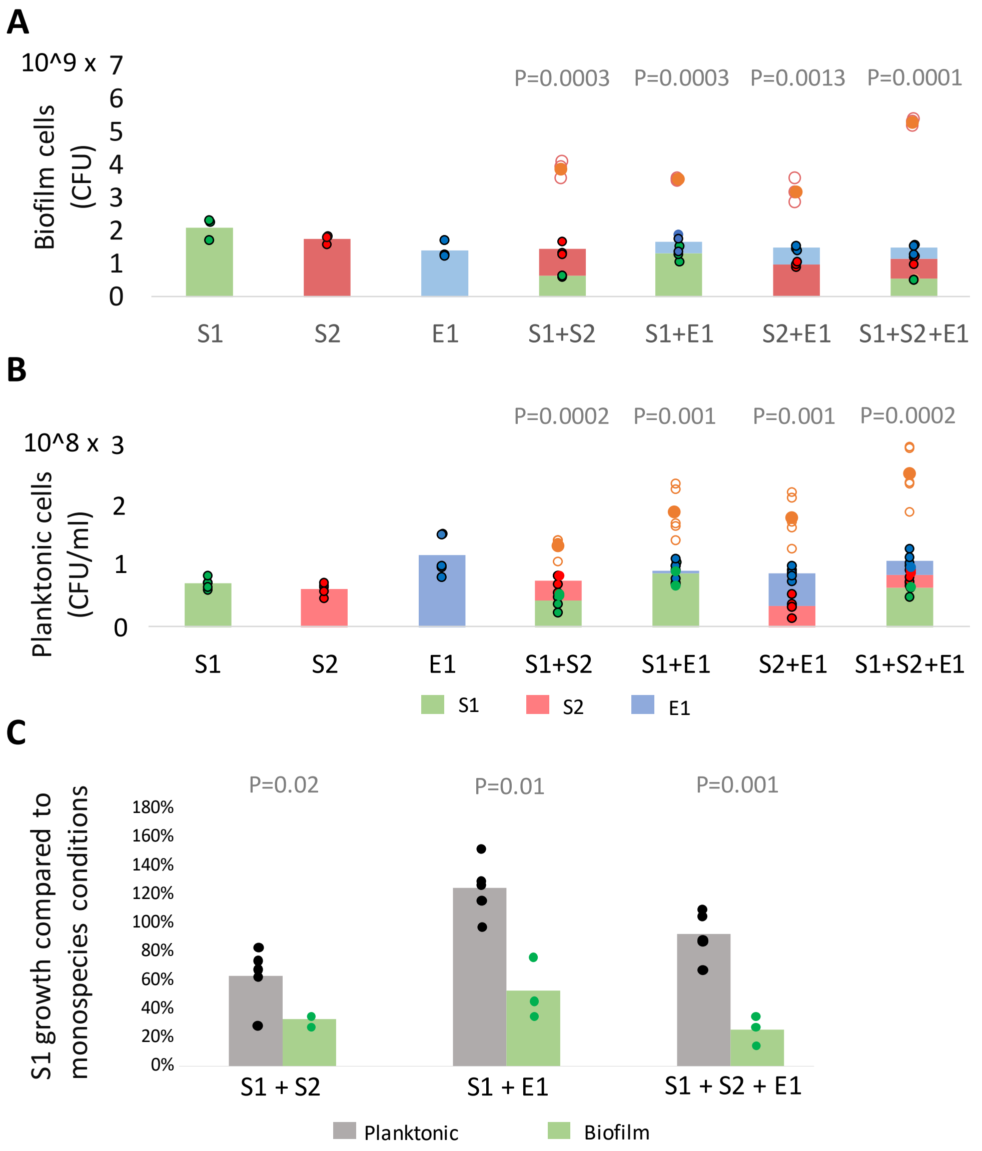

Figure S1. S1 experiences significantly more competition in biofilm conditions than in planktonic conditions, independent of fluorescent marker. Related to Figure 1.

A. Colour swapping does not alter the social interactions in the model community. Cell number of each strain in single-strain, two-strain and three-strain biofilms. The total number of cells expected for cooperation between strains is at minimum equal to the sum of the cells in monoculture and is indicated with orange circles. The interactions are still strongly competitive when plasmids are switched among the strains. S1 was labelled with constitutive dsRed.T4 on a plasmid, while S2 and E1 were labeled with plasmid-encoded constitutive GFPmut3. Differences in colony shape and size allowed differentiation between S2 and E1 during CFU counting. **B.** The interactions are still competitive under planktonic growth conditions. Cell number of each strain in single-strain, two-strain and three-strain planktonic cultures (CFU/ml). The total number of cells expected for cooperation between strains is at minimum the sum of the cells in monoculture and is indicated with orange circles. Five different biological repeats and their average are shown. To differentiate between the strains, S1 was labeled with constitutive GFPmut3 on a plasmid, while S2 and E1 were labeled with plasmid-encoded constitutive dsRed.T4. Differences in colony shape and size allowed differentiation between S2 and E1 during CFU counting **C.** The presence of S2 and E1 inhibits the growth of S1 more in biofilm conditions than in planktonic conditions (absolute data in Figure 1B and Figure S1B). p values are derived from two-tailed student's t-test using Welch's correction if s.d. are significantly ($P < 0.05$) different.

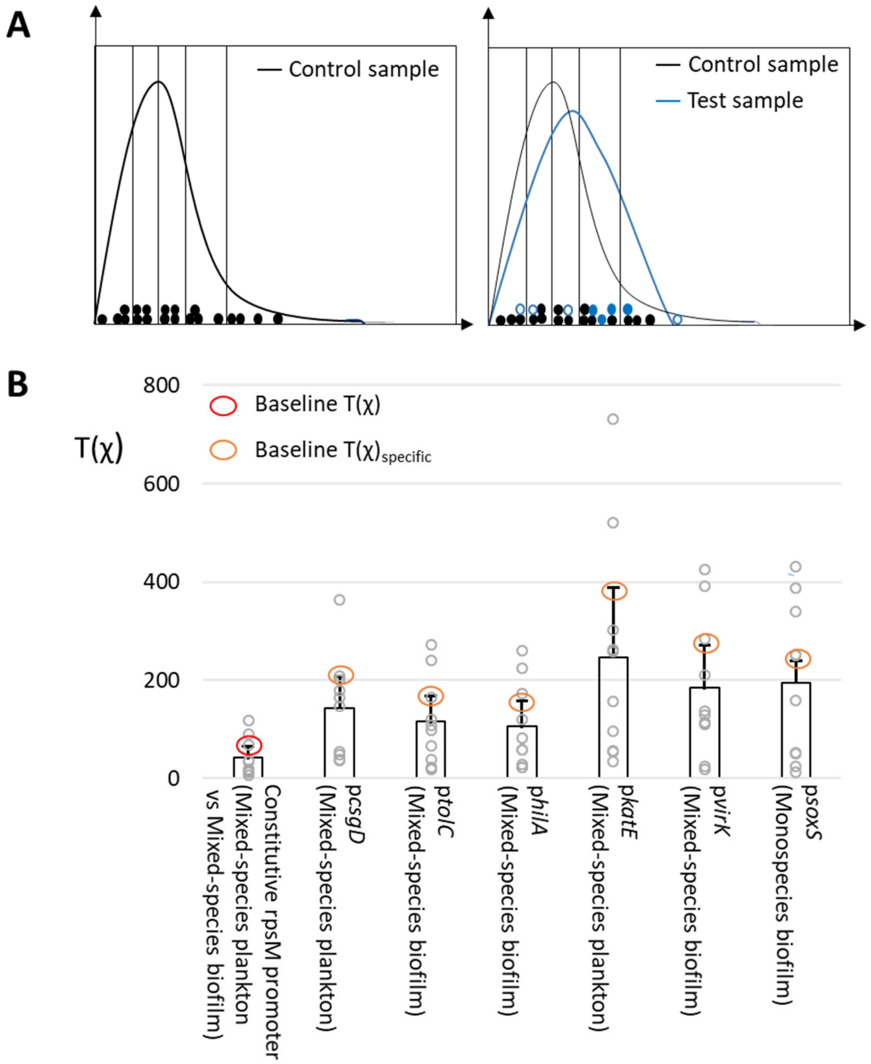

Figure S2. Probability binning identifies significant differences in expression profiles based on a biological relevant threshold value. Related to STAR methods, Figure 2 and Figure 3.

A. Probability binning selects bins such that each bin of the control sample contains the same number of events and subsequently applies these bins to the test sample. The number of events in each corresponding bin is then compared between test and control sample by using a Cox Chi-square test. The Chi Squared value is next conversed to a normalized T(χ) metric that is analogues to a t-score and describes the similarity between two distributions, independent of the number of events or bins. **B**. Determination of baseline $T(\chi)_{\text{minimum}}$ and $T(\chi)_{\text{specific}}$ values that indicate biologically significant differences in gene expression between populations in general or for specific focal genes. T(χ) of the constitutive *rpsM* promoter was repeatedly measured by comparing FACS profiles between mixed-species planktonic and biofilm conditions (n=10). The baseline T(χ) was next determined as the upper limit of the 95% confidence interval of T(χ). A separate T(χ) for each of six focal genes was repeatedly measured for the four test conditions by performing within-condition comparisons (n=10). A baseline $T(\chi)_{\text{specific}}$ was determined for each gene as the upper limit of the 95% confidence interval of the mean of T(χ) in the condition with the highest variation For each reporter gene, the condition with the highest variation and thus the highest $T(\chi)_{\text{specific}}$ is shown.

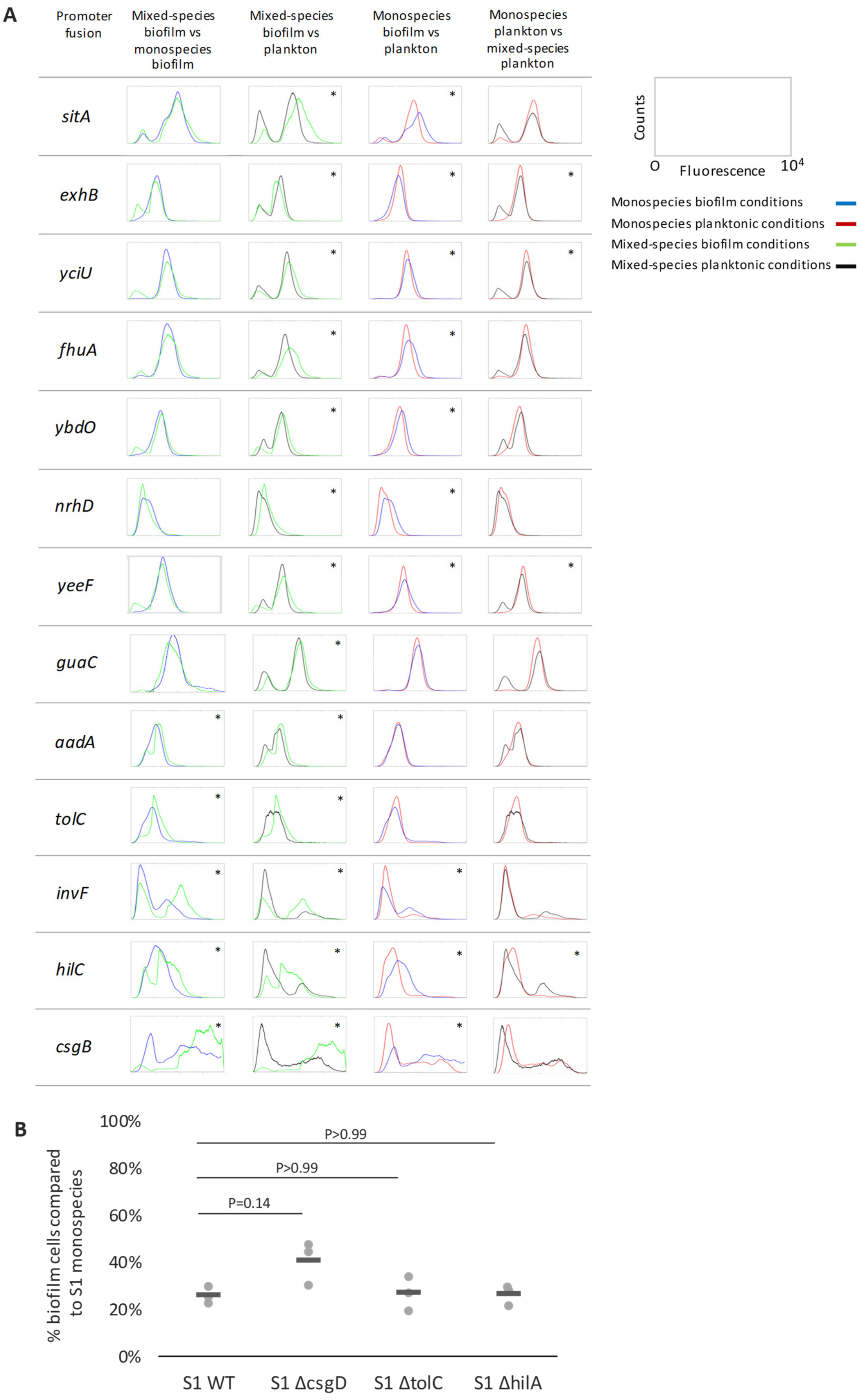
A
Promoter fusion
Mixed-species biofilm vs monospecies biofilm
Mixed-species biofilm vs plankton
Monospecies biofilm vs plankton
Monospecies plankton vs mixed-species plankton
sitA
exhB
yciU
fhuA
ybdO
nrhD
yeeF
guaC
aadA
tolC
invF
hilC
csgB
Counts
0
Fluorescence
10^4
Monospecies biofilm conditions
Monospecies planktonic conditions
Mixed-species biofilm conditions
Mixed-species planktonic conditions
B
% biofilm cells compared to S1 monospecies
100%
80%
60%
40%
20%
0%
P>0.99
P>0.99
P=0.14
S1 WT
S1 ΔcsgD
S1 ΔtolC
S1 ΔhilA

Figure S3. FACS profiles of all 13 genes selected by DFI analysis and subsequent probability binning. Related to Table 1 and Figure 3.

A. Gene expression in SL1344 (S1) was measured by making use of FACS and promoter GFP fusions, under four conditions: monospecies biofilm (blue line), monospecies planktonic (red line), mixed-species biofilm (green line) and mixed-species planktonic (black line) . The FACS profiles show the population distribution of fluorescence in S1 under the different conditions. In each condition, 100 000 S1 cells were analysed. Data were analysed by using the FlowJo software and probability binning. Significant differences between populations ($T(\chi) > T(\chi)$ minimum) are indicated with an asterisk *. One representative repeat of at least two independent biological repeats is shown. **B.** The biofilm cell counts of the S1 wild type and S1 mutants defective in the pathways induced by competition in the presence of competing species. The S1 deletion mutants are not more susceptible to competition than the S1 wild type, confirming that these phenotypes were not solely selected because they allow cells to survive better in mixed-species conditions. p values derived from one-way ANOVA with Bonferroni multiple comparisons correction.

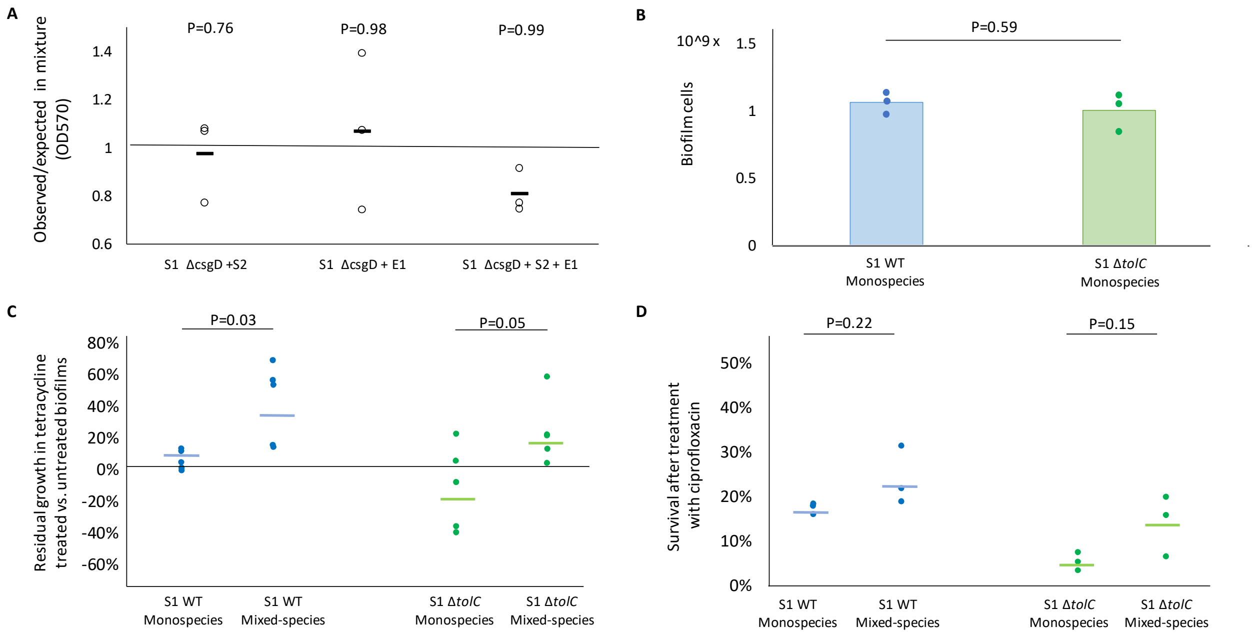

Figure S4. Increased expression of *csgD* in S1 is responsible for the increased matrix production of the mixed-species biofilms whereas the enhanced tolerance to tetracycline is independent of *tolC*. Related to Figure 4.
A. Biomass production is no longer higher than expected if *csgD* is knocked-out in S1. Ratio of the observed amount of biomass (as measured by crystal violet staining) in mixed- species biofilms compared to the expected amount (STAR Methods). p values derived from one sample t-test against 'larger than 1' (n=3). **B.** Deletion of *tolC* does not affect S1 biofilm formation in monospecies conditions. All values are averages of 3 independent biological repeats. P-value derived from two-tailed student's t-test using Welch's correction if s.d. are significantly ($P < 0.05$) different. **C.** Tolerance against tetracycline: in mixed-species conditions, growth is inhibited to a lower extent during treatment with tetracycline compared to monospecies conditions. Additional cellular growth of S1 during 24h incubation of pre-formed monospecies and mixed-species biofilms with fresh growth medium containing 75 µg/ml tetracycline as a percentage compared to the growth in fresh control medium. S1 Δ*tolC* shows an increased susceptibility to tetracycline compared to the wild type in monospecies conditions (the selected concentration even has a slight bactericidal effect). Despite this, knocking out *tolC* does not abrogate the enhanced tolerance in mixed- vs. monospecies biofilms, indicating that the enhanced tolerance is acquired in a TolC-independent manner. All values are averages of 5 independent biological repeats. P-value derived from two-tailed student's t-test using Welch's correction if s.d. are significantly ($P < 0.05$) different. **D**. Tolerance against ciprofloxacin: survival of S1 after 1h incubation of pre-formed monospecies and mixed-species biofilms in the presence of 1 µM ciprofloxacin is higher in mixed-species conditions However, deletion of *tolC* does not abrogate the enhanced tolerance in mixed- vs. monospecies biofilms, indicating that the enhanced tolerance is acquired in a TolC-independent manner. Three biological repeats and their average are shown. P-value are derived from two-tailed student's t-test using Welch's correction if s.d. are significantly ($P < 0.05$) different.

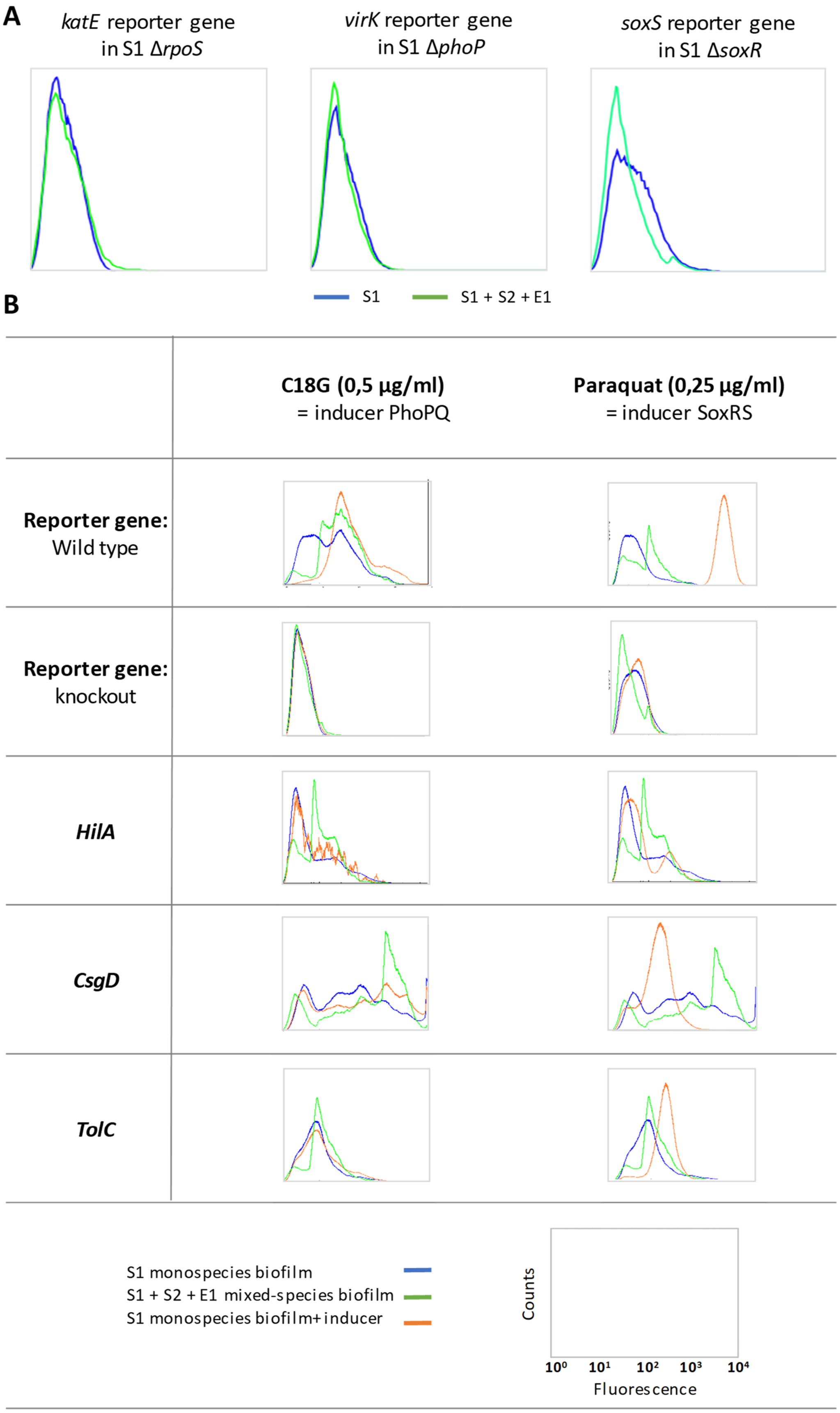
A
katE reporter gene in S1 ΔrpoS
virK reporter gene in S1 ΔphoP
soxS reporter gene in S1 ΔsoxR
S1
S1 + S2 + E1
B
C18G (0,5 µg/ml) = inducer PhoPQ
Paraquat (0,25 µg/ml) = inducer SoxRS
Reporter gene: Wild type
Reporter gene: knockout
HilA
CsgD
TolC
S1 monospecies biofilm
S1 + S2 + E1 mixed-species biofilm
S1 monospecies biofilm+inducer
Counts
10⁰ 10¹ 10² 10³ 10⁴
Fluorescence

Figure S5. Reporter genes and deletion mutants validate the role of stress response systems in the competitive response. Related to Figure 5.

A. *KatE*, *virK* and *soxS* expression reports on the activity of respectively RpoS, PhoPQ and SoxRS since the expression of the reporter genes is abolished when the response systems they report on are deleted. The FACS profiles show the population distribution of fluorescence in S1 mutants under monospecies and mixed-species biofilms. In each condition, a total of 100 000 cells was analysed for their expression behaviour. **B.** Artificial induction of the SoxRS and the PhoPQ stress response systems recapitulates the effect of competition on *csgD*, *hilA* and *tolC*. The effect of C18G on the expression of a reporter gene for PhoPQ (*virK*) was determined in wild type SL1344 (S1) and in an isogenic ΔphoP knockout mutant. Equivalently, the effect of paraquat on the expression of a SoxRS reporter gene (*soxS*) was determined in both the S1 wild type and an isogenic Δ*soxR* knockout mutant. The results confirm that under the tested conditions C18G and paraquat activate the PhoPQ and the SoxRS stress response systems respectively. The expression of *csgD*, *hilA* and *tolC* was determined in S1 by making use of FACS and promoter GFP fusions, under 3 conditions: monospecies biofilm (blue), mixed-species biofilm conditions (green), monospecies biofilm + inducer (orange). The FACS profiles show the population distribution of fluorescence in S1 under the different conditions. In each condition, 100000 cells were analysed for their expression behaviour. Data were analysed by using the FlowJo software and probability binning. Significant differences between monospecies populations with and without inducer ($T(\chi) > T(\chi)$ minimum) are indicated with an asterisk *. One representative repeat of at least two independent biological repeats is shown.

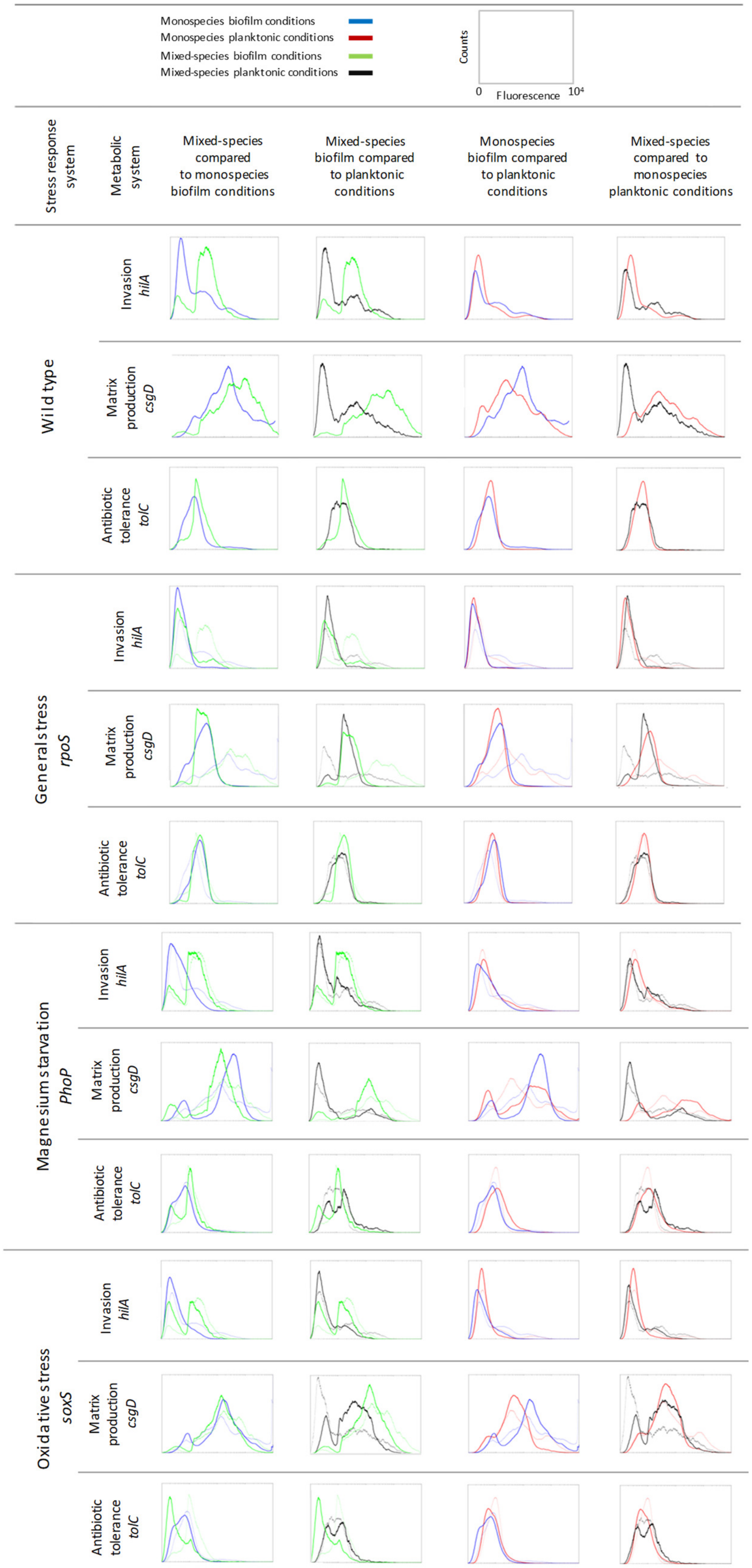
Monospecies biofilm conditions
Monospecies planktonic conditions
Mixed-species biofilm conditions
Mixed-species planktonic conditions
Counts
0
Fluorescence
10^4
Stress response system
Metabolic system
Mixed-species compared to monospecies biofilm conditions
Mixed-species biofilm compared to planktonic conditions
Monospecies biofilm compared to planktonic conditions
Mixed-species compared to monospecies planktonic conditions
Wild type
General stress rpoS
Magnesium starvation PhoP
Oxidative stress soxS
Invasion hilA
Matrix production csgD
Antibiotic tolerance tolC

Figure S6. Overview of the FACS profiles of *csgD*, *hilA* and *tolC* in stress response mutants of *S*. Typhimurium SL1344 (S1). Related to Figure 5.

The expression of *csgD* (matrix production), *hilA* (virulence) and *tolC* (antibiotic tolerance) was determined in wild type S1 and several isogenic stress response mutants by making use of FACS and promoter GFP fusions, under 4 conditions: monospecies planktonic conditions (red), monospecies biofilm conditions (blue), mixed-species planktonic conditions (black) and mixed-species biofilm conditions (green). The FACS profiles show the population distribution of fluorescence in S1 (wild type and mutants) under the different conditions. In each condition, 100000 cells were analysed for their expression behaviour. Data were analysed by using the FlowJo software and probability binning. One representative repeat of at least two independent biological repeats is shown.

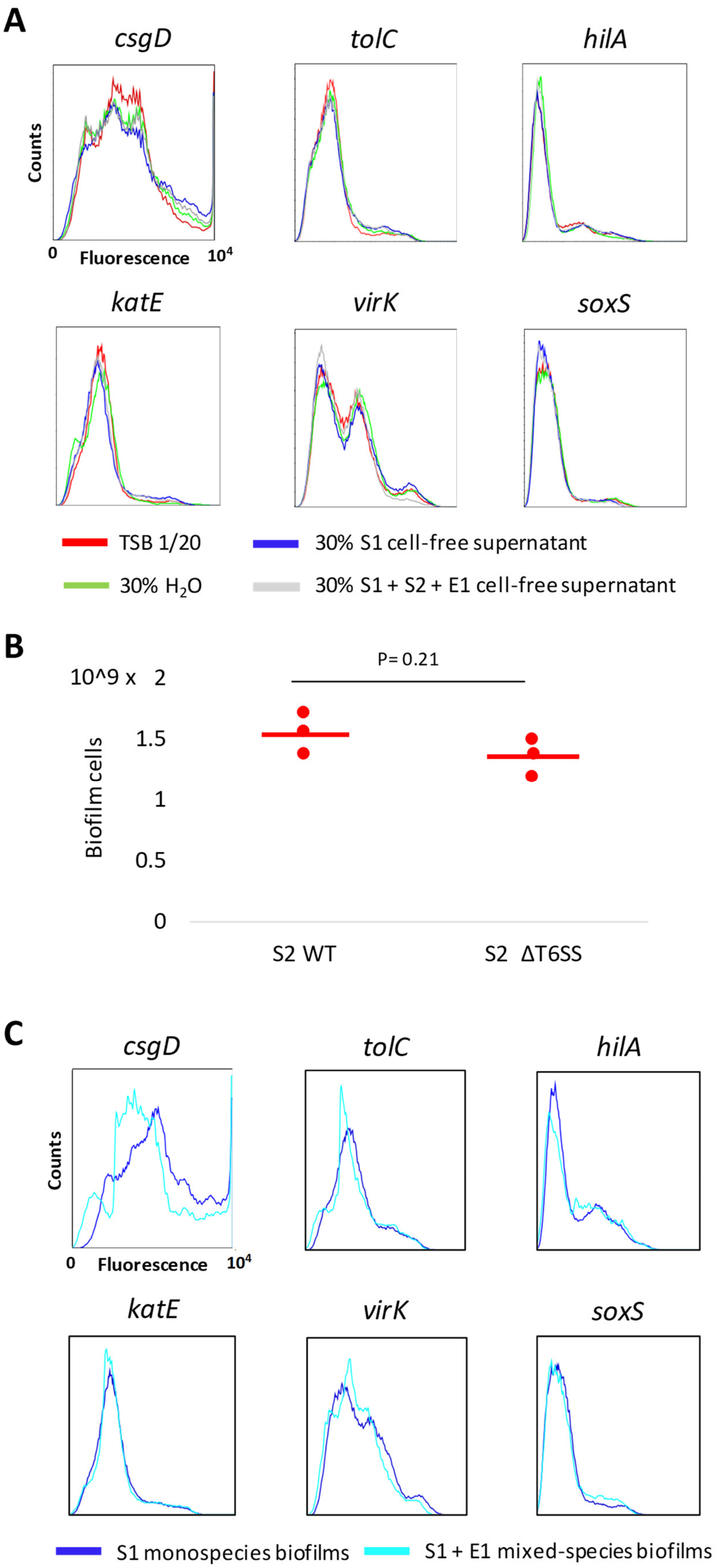
A
csgD
tolC
hilA
Counts
0
Fluorescence
10^4
katE
virK
soxS
TSB 1/20
30% S1 cell-free supernatant
30% H_2O
30% S1 + S2 + E1 cell-free supernatant
B
P= 0.21
10^9 x 2
1.5
1
0.5
0
Biofilm cells
S2 WT
S2 ΔT6SS
C
csgD
tolC
hilA
Counts
0
Fluorescence
10^4
katE
virK
soxS
S1 monospecies biofilms
S1 + E1 mixed-species biofilms

Figure S7. Factors secreted by competitors S2 and E1 or the physical presence of E1 are unable to induce the response to competition in S1. Related to Figure 6.

A. The expression of the reporter genes in a monospecies S1 biofilm grown in the presence of 30% S1 cell-free supernatant, in the presence of 30% S1+S2+E1 cell-free supernatant or in growth medium diluted with 30% H_2O was measured. The FACS profiles show the population distribution of fluorescence in S1 under the different conditions. In each condition, 100 000 S1 cells were analysed. Data were analysed by using the FlowJo software. Probability binning could not detect significant differences between populations. However, the cell free-supernatant collected from the mixed-species biofilm seems to slightly induce the expression of *csgD*, *tolC* and *katE,* albeit to a much lower extent than in the physical presence of competitors. A control containing 30% water instead of supernatant also slightly induces the expression of *csgD* and *tolC*, but to an even lower extent than the cell-free supernatant, indicating that nutrient depletion alone cannot fully explain the effect of the cell-free supernatant. Remarkably, the cell-free supernatant of the monospecies S1 matched (*tolC*, *katE*) or exceeded (*csgD*) the induction by the mixed-species supernatant. This indicates that disrupted administration of factors secreted by S1, and not secreted competitive factors of S2 and E1, is likely responsible for the gene induction by the mixed species supernatant. One representative repeat of at least two independent biological repeats is shown. **B.** The S2 ΔT6SS mutant does not have a significantly lower number of cells in the biofilm than the S2 wild type. p values are derived from two-tailed student's t-test using Welch's correction if s.d. are significantly ($P < 0.05$) different (n=3). **C.** The expression of the reporter genes in S1 if cultured in monospecies conditions or in the presence of E1 alone was measured. E1 has does not increase the expression of the reporter genes in S1. The FACS profiles show the population distribution of fluorescence in S1 under the different conditions. In each condition, a total of 100 000 S1 cells was analysed. Data were analysed by using the FlowJo software and probability binning. Significant differences ($T(\chi) > T(\chi)$ specific) are indicated with an asterisk *. One representative repeat of at least two independent biological repeats is shown.

Primer	Sequence 5' → 3'	Purpose
PRO 4	GTGCCACCTGACGTCTAAGAAACC	FW, sequencing pFPV25
PRO 0406	CATATGTATATCTCCTTCTTAAATCTAG	RV, sequencing pFPV25
PRO 5218	TGCTCTCTCCGAACATTAAGGTGTA	FW, amplification of *sitA* promoter region
PRO 5219	TCGCGACAATACCGGCAATCAGG	RV, amplification of *sitA* promoter region
PRO 8898	ATCCCGGGGCAGTAAAGGTGCGGCAGTATGT	FW, amplification of *exbB* promoter region
PRO 8899	ATTCTAGATCGGCGTGCTGATACATGCCC	RV, amplification of *exbB* promoter region
S&P-00281	ATCCCGGGAAAACGTCCGTTATGGCAGC	FW, amplification of *yciU* promoter region
S&P-00282	ATTCTAGAGCCGCCAGCTCAAGAAAAAT	RV, amplification of *yciU* promoter region
PRO 5222	GGCGCGATGGCGATTTACCA	FW, amplification of *fhuA* promoter region
PRO5223	CAGACATGCCGCTAACCGCT	RV, amplification of *fhuA* promoter region
S&P-00295	ATTCTAGAAGATACATTCAAATATAACCAACAGGT	FW, amplification of *ybdO* promoter region
S&P-00296	ATCCCGGGGGAAAAAGCGCAACTGGACG	RV, amplification of *ybdO* promoter region
PRO 5530	ATCCCGGGCAGCGGCGTTAAAATCAATCGAAAATT CCC	FW, amplification of *nrdH* promoter region
PRO 5531	ATTCTAGAGGCACTGAACACAGTTATTGCGAGTGT AAA	RV, amplification of *nrdH* promoter region
S&P-00297	ATTCTAGACGCGAGAGGTGTTTGGAGTA	FW, amplification of *yeeF* promoter region
S&P-00298	ATCCCGGGGGGGTGTATGCAAAATCGCC	RV, amplification of *yeeF* promoter region
S&P-00235	ATCCCGGGTGCCAATACCGCCGGTTAAG	FW, amplification of *guaC* promoter region
S&P-00236	ATTCTAGAGGGTAGAACGCTTAGGACGG	RV, amplification of *guaC* promoter region
S&P-00243	ATCCCGGGAGGCGAATGAGGTCGTTGAC	FW, amplification of *aadA* promoter region
S&P-00244	ATTCTAGACGCTTCCGTTTGGCATTGAA	RV, amplification of *aadA* promoter region
S&P-00255	ATTCTAGAGGTCTCCTGATACTGGTGCG	FW, amplification of *invF* promoter region
S&P-00256	ATCCCGGGAGAAGAATGAGGCGCCATGT	RV, amplification of *invF* promoter region
S&P-00259	ATTCTAGATGTTCGGCTGTTGAAGGTGA	FW, amplification of *hilC* promoter region
S&P-00260	ATCCCGGGTATCGGGCGGGGAAAACAAA	RV, amplification of *hilC* promoter region

PRO 0404	GGAGGATCCAGATCATAATTTGTCG	FW, amplification of *csgB* promoter region
PRO 0563	GGAGGATCCAGCTTCTTATCCGCTTCCATCATATCC	RV, amplification of *csgB* promoter region
S&P-00273	ATCCCGGGCGCGTTTCCCGTGCAATAAT	FW, amplification of *tolC* promoter region
S&P-00274	ATTCTAGAGCTCAGGCCGATAAGGATGG	RV, amplification of *tolC* promoter region
S&P-01402	ATCCCGGGCACCAGAATTATCAAAGCGGCA	RV, amplification of *lexA* promoter region
S&P-01403	ATTCTAGATCTTGTTGCCTGGCCGTTAA	RV, amplification of *lexA* promoter region
S&P-01394	ATCCCGGGCAGCTCCGGGATCTGGTTTT	FW, amplification of *micA* promoter region
S&P-01395	ATTCTAGATGGCCAAAATTTCATCGCTGA	RV, amplification of *micA* promoter region
S&P-01398	ATCCCGGGTGGCAGGGAAATGTCCTACG	FW, amplification of *omrB* promoter region
S&P-01399	ATTCTAGAAACGTCATTGTTGATCACACGA	RV, amplification of *omrB* promoter region
S&P-01101	ATCCCGGGCTTTGCTTTCATAGAAGTGCAGGG	FW, amplification of *soxS* promoter region
S&P-01108	ATTCTAGACGCTGCAAATACCACTTGG	RV, amplification of *soxS* promoter region
S&P-01404	ATCCCGGGTGCGTATTAACCACGGCGAT	FW, amplification of *feoB* promoter region
S&P-01405	ATTCTAGAGTTTTTGACGATACGCCGGG	RV, amplification of *feoB* promoter region
PRO 6290	ATCCCGGGTCCCCGCGCCAAAAGCAGAT	FW, amplification of *virK* promoter region
PRO 6291	ATTCTAGAGGCGTCTGCGCCATGACTTCT	RV, amplification of *virK* promoter region
S&P-01396	ATCCCGGGGCGTGGTTTATCTGCCATGC	FW, amplification of *oxyS* promoter region
S&P-01397	ATTCTAGATAACCCTTGAAGACACCGCC	RV, amplification of *oxyS* promoter region

Table S1. Primers used throughout this study. Related to STAR Methods.

**Plasmids**	**Source**	**Description**
pFPV25	[S1]	Promoter-trap vector constructed by inserting an EcoRI-HindIII fragment containing a promoterless *GFPmut3* into plasmid pED350 (colE1, *bla*, *mob*); Ap^R
pFPV25.1_GREEN	[S1]	0.6 kb *Sau3AI* fragment inserted in the BamHI site of pFPV25, containing the promoter region of S. Typhimurium *rpsM* encoding for the ribosomal protein S13 (constitutive promoter); Ap^R
pFPV25.1_RED	This work	Identical to pFPV25.1_GREEN; *GFPmut3* gene replaced by *dsRed*.T4 gene
pCMPG5521	[S2]	pFPV25 plasmid with the promoter region of *csgD* inserted in front of the *GFPmut3* gene
pCMPG10105	[S2]	pFPV25 plasmid with the promoter region of *sitA* inserted in front of the *GFPmut3* gene
pCMPG10120	This work	pFPV25 plasmid with the promoter region of *exbB* inserted in front of the *GFPmut3* gene
pCMPG10145	This work	pFPV25 plasmid with the promoter region of *yciU* inserted in front of the *GFPmut3* gene
pCMPG10116	[S3]	pFPV25 plasmid with the promoter region of *fhuA* inserted in front of the *GFPmut3* gene
pCMPG10152	This work	pFPV25 plasmid with the promoter region of *ybdO* inserted in front of the *GFPmut3* gene
pCMPG10110	[S3]	pFPV25 plasmid with the promoter region of *nrdH* inserted in front of the *GFPmut3* gene
pCMPG10153	This work	pFPV25 plasmid with the promoter region of *yeeF* inserted in front of the *GFPmut3* gene
pCMPG10123	This work	pFPV25 plasmid with the promoter region of *guaC* inserted in front of the *GFPmut3* gene
pCMPG10127	This work	pFPV25 plasmid with the promoter region of *aadA* inserted in front of the *GFPmut3* gene
pCMPG10132	This work	pFPV25 plasmid with the promoter region of *invF* inserted in front of the *GFPmut3* gene
pCMPG10134	This work	pFPV25 plasmid with the promoter region of *hilC* inserted in front of the *GFPmut3* gene
pCMPG5539	[S2]	pFPV25 plasmid with the promoter region of *csgB* inserted in front of the *GFPmut3* gene
pCMPG10169	This work	pFPV25 plasmid with the promoter region of *tolC* inserted in front of the *GFPmut3* gene
pCMPG10021	[S3]	pFPV25 plasmid with the promoter region of *katE* inserted in front of the *GFPmut3* gene
pCMPG10104	[S3]	pFPV25 plasmid with the promoter region of *cpxP* inserted in front of the GFPmut3 gene
pCMPG11400	This work	pFPV25 plasmid with the promoter region of *feoB* inserted in front of the GFPmut3 gene
pCMPG11401	This work	pFPV25 plasmid with the promoter region of *omrB* inserted in front of the GFPmut3 gene
pCMPG5547	[S2]	pFPV25 plasmid with the promoter region of *sspA* inserted in front of the GFPmut3 gene
pCMPG11402	This work	pFPV25 plasmid with the promoter region of *oxyS* inserted in front of the GFPmut3 gene

pCMPG11403	This work	pFPV25 plasmid with the promoter region of *lexA* inserted in front of the GFPmut3 gene
pCMPG11405	This work	pFPV25 plasmid with the promoter region of *micA* inserted in front of the GFPmut3 gene
pCMPG11406	This work	pFPV25 plasmid with the promoter region of *virK* inserted in front of the *GFPmut3* gene
pCMPG11407	This work	pFPV25 plasmid with the promoter region of *soxS* inserted in front of the *GFPmut3* gene
pKD4	[S4]	Plasmid used as template for the chloramphenicol resistance cassette flanked by FLP recognition target sites for the construction of *Salmonella* knock-out mutants
pKD46	[S4]	Lambda Red helper plasmid
pCP20	[S4]	FLP recombinase helper plasmid

Table S2. Plasmids used throughout this study. Related to STAR Methods.

Supplemental References

S1. Valdivia, R.H., and Falkow, S. (1996). Bacterial genetics by flow cytometry: rapid isolation of *Salmonella typhimurium* acid-inducible promoters by differential fluorescence induction. Mol. Microbiol. *22*, 367-378.

S2. Hermans, K., Nguyen, T.L.A., Roberfroid, S., Schoofs, G., Verhoeven, T., De Coster, D., Vanderleyden, J., and De Keersmaecker, S.C. (2011). Gene expression analysis of monospecies *Salmonella* Typhimurium biofilms using Differential Fluorescence Induction. J. Microbiol. Methods *84*, 467-478.

S3. Robijns, S.C., Roberfroid, S., Van Puyvelde, S., De Pauw, B., Uceda Santamaria, E., De Weerdt, A., De Coster, D., Hermans, K., De Keersmaecker, S.C., Vanderleyden, J., et al. (2014). A GFP promoter fusion library for the study of *Salmonella* biofilm formation and the mode of action of biofilm inhibitors. Biofouling *30*, 605-625.

S4 Datsenko, K.A., and Wanner, B.L. (2000). One-step inactivation of chromosomal genes in *Escherichia coli* K-12 using PCR products. PNAS *97*, 6640-6645.
